# Quantitative Resistance to *Verticillium* Wilt in *Medicago truncatula* Involves Eradication of the Fungus from Roots and Is Associated with Transcriptional Responses Related to Innate Immunity

**DOI:** 10.3389/fpls.2016.01431

**Published:** 2016-09-29

**Authors:** Maoulida Toueni, Cécile Ben, Aurélie Le Ru, Laurent Gentzbittel, Martina Rickauer

**Affiliations:** ^1^EcoLab, Université de Toulouse, CNRS, INPT, UPSToulouse, France; ^2^Research Federation “Agrobiosciences, Interactions et Biodiversité”Castanet-Tolosan, France

**Keywords:** gene co-expression analysis, hormone signaling, legumes, PAMP-triggered immunity, quantitative disease resistance, root disease, soil-borne pathogens, transcriptomics

## Abstract

Resistance mechanisms to *Verticillium* wilt are well-studied in tomato, cotton, and Arabidopsis, but much less in legume plants. Because legume plants establish nitrogen-fixing symbioses in their roots, resistance to root-attacking pathogens merits particular attention. The interaction between the soil-borne pathogen *Verticillium alfalfae* and the model legume *Medicago truncatula* was investigated using a resistant (A17) and a susceptible (F83005.5) line. As shown by histological analyses, colonization by the pathogen was initiated similarly in both lines. Later on, the resistant line A17 eliminated the fungus, whereas the susceptible F83005.5 became heavily colonized. Resistance in line A17 does not involve homologs of the well-characterized tomato *Ve*1 and *V. dahliae Ave*1 genes. A transcriptomic study of early root responses during initial colonization (i.e., until 24 h post-inoculation) similarly was performed. Compared to the susceptible line, line A17 displayed already a significantly higher basal expression of defense-related genes prior to inoculation, and responded to infection with up-regulation of only a small number of genes. Although fungal colonization was still low at this stage, the susceptible line F83005.5 exhibited a disorganized response involving a large number of genes from different functional classes. The involvement of distinct phytohormone signaling pathways in resistance as suggested by gene expression patterns was supported by experiments with plant hormone pretreatment before fungal inoculation. Gene co-expression network analysis highlighted five main modules in the resistant line, whereas no structured gene expression was found in the susceptible line. One module was particularly associated to the inoculation response in A17. It contains the majority of differentially expressed genes, genes associated with PAMP perception and hormone signaling, and transcription factors. An *in silico* analysis showed that a high number of these genes also respond to other soil-borne pathogens in *M. truncatula*, suggesting a core of transcriptional response to root pathogens. Taken together, the results suggest that resistance in *M. truncatula* line A17 might be due to innate immunity combining preformed defense and PAMP-triggered defense mechanisms, and putative involvement of abscisic acid.

## Introduction

Plants continuously have to cope with attacks from pathogens or pests. Although in most cases these attacks are efficiently encountered by the plants' natural defense mechanisms, plant disease is still a major constraint in agricultural productivity. Breeding for pathogen resistance to answer a growing demand for food supply while reducing pesticide use, needs in-depth understanding of plant disease resistance mechanisms.

Plant innate immunity is an active defense system taking place after a pathogen had overcome preformed defenses. The perception of conserved microbial molecular signatures (pathogen-associated molecular patterns, PAMPs) by plant receptors then initiates signaling cascades and transcription reprogramming leading to the so-called PAMP-triggered immunity (PTI; Zipfel, 2014). Main responses driven by PTI are the synthesis of antimicrobial compounds and pathogenesis-related (PR) proteins. However, this innate immunity can be inactivated by adapted pathogens which secrete effector molecules which interrupt the signal transduction leading to PTI. A co-evolutionary arms race between host plants and pathogens has led to a second layer of plant defense called effector-triggered immunity (ETI), which relies on direct or indirect recognition of pathogen effectors by plant intracellular resistance (R) proteins (Dangl et al., 2013). ETI, which is conditioned by a single R gene typically yields complete (qualitative) disease resistance against pathogens containing the recognized effector. It is thus specific to pathogen race and easily overcome by the evolution of new races (Dangl et al., 2013).

In contrast to qualitative resistance, quantitative disease resistance (QDR) is characterized by a continuous range of phenotypes within segregating or natural populations (Poland et al., 2009). It is conditioned by multiple genes of sometimes small effect (Quantitative Trait Loci, QTL) which may further interact with the environment (Roux et al., 2014) and may lead to total absence of symptoms in genotypes gathering all favorable alleles. QDR is not specific of pathogen race offering thus a broader spectrum, and due to its polygenic inheritance it is presumably more durable (Roumen, 1994). Genes controlling QDR have been cloned in several species. Only a few resemble classical Nucleotide-Binding Site Leucine Rich Repeat (NBS-LRR) R-genes; most were previously unidentified genes, with biological functions not formerly associated with disease resistance (reviewed in Roux et al., 2014; Hurni et al., 2015; Debieu et al., 2016). These first insights highlight the diversity of molecular mechanisms triggering QDR which integrates multiple perception pathways, each contributing to the overall resistance phenotype. Better understanding of its cellular and molecular regulation networks is required to develop strategies for long-term broad-spectrum control of plant diseases.

*Verticillium* wilt disease, caused by soil-borne fungi of the genus *Verticillium* is a major constraint for production of important crops, such as tomato or cotton (Fradin and Thomma, 2006; Klosterman et al., 2009). After entering the root through wounds or cracks at the site of lateral root emergence, the fungus colonizes the xylem vessels, which, together with gum formation by plant cells, induces vessel clogging and results in the typical wilting symptoms (Agrios, 2004). Due to survival structures viable for many years in the soil and to the protected localization in infected plants, *Verticillium* wilt is difficult to control. So far the most efficient way is by breeding resistant varieties.

Examples of polygenic and monogenic *Verticillium* resistance have been described and resistance loci have been identified (e.g., Simko et al., 2004; Wang et al., 2008; Yang C. et al., 2008; Häffner et al., 2010; Zhang et al., 2012; Jakse et al., 2013). Notably the *Ve* locus in tomato which confers resistance to race 1 of *V. dahliae* and *V. albo-atrum* (Fradin and Thomma, 2006) has been characterized in detail. It contains two genes encoding receptor-like proteins with NBS-LRR domains, *Ve1* and *Ve2*, which are each able to confer resistance to susceptible potato (Kawchuk et al., 2001). In Arabidopsis, the tomato *Ve1* gene confered resistance to *V. dahliae* and *V. albo-atrum* but not to *V. longisporum* (Fradin et al., 2011). Homologs of the *Ve* genes have been reported in other species as well (Fradin and Thomma, 2006).

Defense mechanisms against *Verticillium spp*. and their control by phytohormones and other signaling pathways have been studied in several hosts, such as tomato, cotton, or Arabidopsis (Gayoso et al., 2010; Zhang Y. et al., 2013; Köenig et al., 2014). In most cases, resistant plants are characterized by a rapid increase in phenylalanine ammonia lyase (PAL) activity, an enzyme involved in the synthesis of lignin and phenylpropanoids and in synthesis of salicylic acid (SA), an important signaling compound in resistance (Mauch-Mani and Slusarenko, 1996). Data on the role of most phytohormones in resistance to *Verticillium* are however contradictory. A given hormone might be linked either to resistance (Hu et al., 2005; Johansson et al., 2006; Fradin et al., 2009, 2011) or to symptom development (Tjamos et al., 2005; Ratzinger et al., 2009; Häffner et al., 2014; Roos et al., 2014), depending on plant species and experimental approaches.

Legume plants have an essential role in sustainable agriculture, due to their symbiosis with nitrogen-fixing rhizobacteria and the high protein content of their seeds. Their roots are thus a site of sometimes simultaneous responses to symbiotic and pathogenic microbes. *Verticillium* wilt, caused by *V. alfalfae* (*Va*), is a serious threat to the forage legume crop alfalfa, notably in Europe. Although tolerant alfalfa cultivars are available, the bases of resistance or tolerance in this autotetraploid and allogamous species are not well-understood (Molinéro-Demilly et al., 2006; Zhang et al., 2014; Yu et al., 2016). The closely related wild species *Medicago truncatula* is a model for legume plants and particularly attractive for the study of plant-microbe interactions (Samac and Graham, 2007; Rose, 2008; Young and Udvardi, 2009; Gentzbittel et al., 2015). Knowledge obtained with *M. truncatula* can be transferred to alfalfa and other legumes. For example, the *RCT1* gene responsible for resistance against *C. trifolii* race 1 in *M. truncatula*, conferred resistance against several races of the fungus in alfalfa (Yang S. et al., 2008). *M. truncatula* is also prone to *Verticillium* wilt and exhibits a high biodiversity in the response to this pathogen (Ben et al., 2013; Negahi et al., 2013). Genetic analyses performed on different crosses revealed that *Verticillium* wilt response in *M. truncatula* is a QDR, regulated by QTL that differ across resistant accessions and according to the fungus strains (Ben et al., 2013; Negahi et al., 2014). None of these QTL are co-localized with putative *Ve* homologs. The highly contrasted phenotypes of resistant line A17 and susceptible F83005.5 as well as the fact that resistance in A17 is controlled by one major QTL makes this couple a good model to explore resistance and defense mechanisms against *Verticillium* wilt, and more generally to gain knowledge on molecular mechanisms involved in QDR. Hence, these two lines were used to characterize the resistance response by microscopy and a transcriptomics-based approach.

## Materials and methods

### Plants

*M. truncatula* A17 and F83005.5 plants were grown in hydroponic culture on Farhaeus medium as described by Ben et al. (2013), with 16 h of light (170 μmol m^−2^ s^−1^) at 25°C and 8 h of darkness at 23°C.

### Fungal isolates

*V. alfalfae* V31-2 (formerly *V. albo-atrum*), its GFP-expressing transformant *Va*-A1b2 (Ben et al., 2013), *V. albo-atrum* LPP0323 (provided by A. v. Tiedemann, Germany), and *V. dahliae* JR2 (provided by B. Thomma, Netherlands) were grown on PDA medium at 24°C in the dark. Spore suspensions were obtained as described in Ben et al. (2013).

### Inoculation and symptom scoring

Ten day old plants were inoculated after cutting the root apex by root-dipping in a conidial suspension (10^6^ sp/ml) for 60 min. Then roots were kept in sterile water for 10 min, transferred back to nutritive solution and incubated in a growth chamber at 20°C with 16 h photoperiod. Symptoms were scored on a scale from 0 (healthy) to 4 (dead plant) (Supplementary Figure S1B). Area Under the Disease Progress Curves (AUDPC, Shaner and Finney, 1977) were computed using the “agricolae” package of the R system for statistical computing and graphing (R Core Team, 2012). All data were analyzed with ANOVA using the appropriate model depending on the experimental designs used. When required, data transformations were applied to achieve normality and homoscedasticity of ANOVA residuals. Pairwise treatment differences were determined by a Tukey's test or Newman-Keuls test using the “agricolae” package.

### Microscopic observations

*M. truncatula* roots were rinsed briefly in water and 1 cm fragments were embedded in 5% low melting point agarose. Longitudinal sections of 45 μm were prepared on a vibratom (Leica VT 1000S) and mounted in distilled water. Confocal images were acquired with a spectral confocal laser scanning system (SP2 SE, Leica) equipped with an upright microscope (DM 6000, Leica, Germany). Phenolic compounds were observed using an inverted microscope (Leica DM IRBE, Leica) under UV light. Images were acquired with a CCD camera (Color Coolview; Photonic Science) using an objective with 40 × magnification.

### Gene expression analysis by qRT-PCR

Total RNA was extracted using TRIzol reagent (Invitrogen) following the manufacturer's instructions. After treatment with RQ1 RNase-Free DNase (Promega) cDNA was synthesized using the ImProm-II™Reverse Transcription System (Promega). Quantitative PCR (qPCR) was carried out with standard protocol (see protocol details in Supplementary Figure S4) using 3 μl of cDNA (3 ng/μl).

Data analysis was performed with the SDS 2.3 software (Applied Biosystems). C_T_-values were normalized against the harmonic mean of four reference genes Medtr2g099090.1, Medtr2g033910.1, Medtr3g085850.1, and TC117750 (encoding Glyoxalase 2, Phosphatase 2C, G3PDH, and H3L, respectively). Primers are shown in Supplementary Table S1.

Fold change in gene expression was determined with the ΔΔC_T_ method, using the mock-inoculated condition as the reference (Livak and Schmittgen, 2001).

### Transcriptomic analysis by massive analysis of cDNA ends (MACE)

Six roots of *Va*- and mock-inoculated A17 and F83005.5 plants were harvested at t0, 4, 8, and 24 hpi, and shock-frozen in liquid nitrogen. Total RNA was extracted with TRIzol reagent (Invitrogen). Samples from 4, 8, and 24 hpi were equimolarily pooled for mock-inoculated or *Va*-inoculated plants. Twenty μg RNA were processed for Massive Analysis of cDNA Ends.

MACE was conducted by GenXPro GmbH as described in Behringer et al. (2015). Tag assembly and annotation were conducted by GenXPro GmbH. After sequencing, each 93 nt tag was mapped allowing only 1 mismatch to sequences in databases including *M. truncatula* genome version 3.5v5 and *M. truncatula* ESTs of MtGI Release 11.0.

### Gene expression profiling

Differential expression analysis was conducted using “edgeR” package of the R/Bioconductor statistical language (Robinson et al., 2010). Reads non-uniquely aligned and in incorrect orientation with respect to genome annotation were discarded. Genes with ≥5 reads were retained.

Normalized read counts were obtained by Trimmed Mean of M-values (TMM) normalization and were modeled by a negative binomial distribution, which allows over-dispersion of counts, and fitted using generalized linear models to test for differential abundances (i) between A17 and F83005.5 “t0” libraries to characterize putative genotype effect on gene expression before inoculation (ii) between mock- and *Va*-inoculated libraries to detect genes responding to inoculation in each genotype. Biological replicate was fitted as an effect. Differential expression was tested in “tag-wise” mode, where the dispersion is set to the observed dispersion for the transcripts. Logistic regression of the transcript proportions was also used (Collett, 2002). *P-values* were adjusted to control the false discovery rate using the Benjamini-Hochberg method.

### Construction of co-regulated gene networks using WGCNA and LegumeGRN software

Weighted gene co-expression networks were constructed using the “WGCNA” R package (Langfelder and Horvath, 2008). Transcripts with a coefficient of variation (CV) ≥ 0.35 for normalized read counts across the six libraries from the A17 resistant line were selected. This CV threshold was chosen to include all A17 differentially expressed genes (DEGs) responsive to inoculation in the resulting trimmed dataset. WGCNA was performed using similar settings as in Formey et al. (2014) except a beta power of 20. Eigengene value was calculated for each identified module and used to test for association with different contrasts of biological interest (i.e., “mock-inoculated vs. *Va*-inoculated roots”) and contrasts accounting for the effect of the biological replicates. The five modules most correlated to inoculation response were visualized using Cytoscape v3.0.1 (Smoot et al., 2011).

Genes from the Greenyellow module possessing Affymetrix probes were analyzed using the legumeGRN gene regulatory network prediction server (http://legumegrn.noble.org/, Wang M. et al., 2013). Unweighted Gene Co-expression Network was predicted using compiled data from the *M. truncatula* Gene Expression Atlas (MtGea) regarding different pathogenic root interactions. A Pearson's correlation threshold of |0.7| was applied.

### *In silico* functional analyses

#### GO-term enrichment analysis

Gene Ontology (GO) terms were generated using standard settings of Blast2GO (Conesa et al., 2005; http://www.blast2go.com). GO term enrichment analysis was performed with the Singular Enrichment Analysis (SEA) tool of agriGo (Du et al., 2010) using the “complete GO” set, Blast2GO-customized annotation for the test sample and “*M. truncatula* genome locus v3.5” as background reference. Statistical tests were done with Fisher's exact test and *p*-values were adjusted with Yekutieli (FDR under dependency) multi-test correction.

#### MapMan analysis

To create the mapping files necessary to visualize MACE data using MapMan software (Thimm et al., 2004), *M. truncatula* transcripts were first assigned to their respective MapMan BIN from the existing pathway mapping file for *M. truncatula* genome v3.5 sequences (Mt_Mt3.5_v3_0411.m02). Remaining genes were annotated using InterProscan 4.8 for accurate annotation then classified manually according to their respective TIGR annotation (TAIR 7.0) on The GABI Primary Database (http://www.gabipd.org/).

## Results

### *Medicago truncatula* line A17 eliminates *V. alfalfae* from roots after initial colonization

A hydroponic culture which allows easy access to roots as described by Ben et al. (2013) was used in this work. After inoculation with *V. alfalfae* (*Va*) strain V31-2, the susceptible line F83005.5 developed disease symptoms and aerial fresh biomass was reduced at the end of the experiment, whereas the resistant line A17 was not affected (Supplementary Figure S1). To assess if this difference was correlated to root colonization, both lines were inoculated with *Va*-A1b2, a GFP-expressing strain, and root sections were observed at different times after inoculation using confocal laser scanning microscopy.

**Figure 1:**
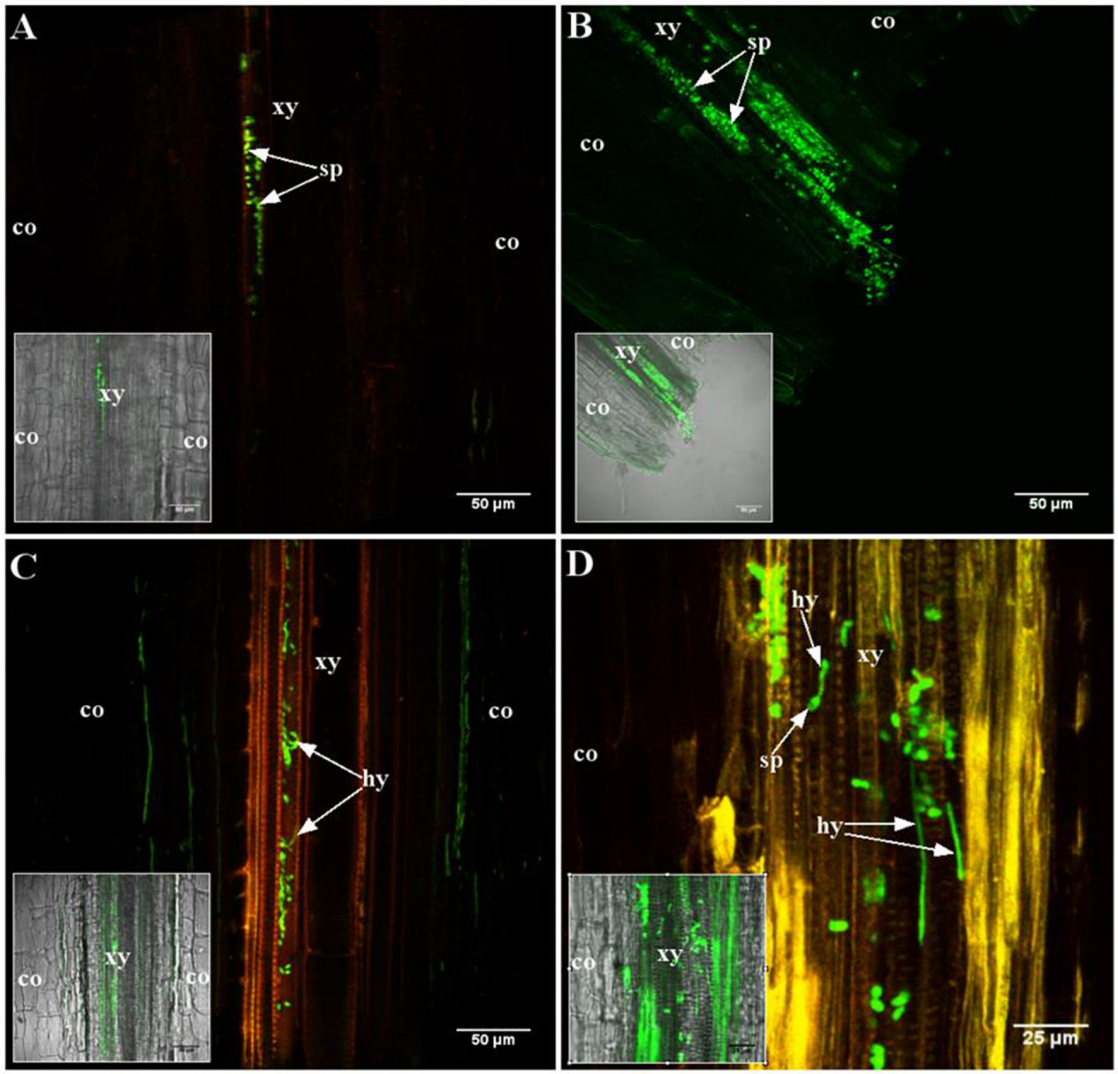
**Early stages of root colonization of A17 (resistant) and F83005.5 (susceptible) by a GFP-expressing strain of *Va* V31-2**. Observations were made on 45 μm thick longitudinal root sections embedded in 5% agarose. Confocal images were acquired with a spectral confocal laser scanning system (SP2 SE, Leica, Germany) equipped with an upright microscope (DM 6000, Leica, Germany). Observations were made using 10 × (HC PL Fluotar, N.A. 0.3) and 40 × (HCX PL APO, N.A. 0.8) dry and water immersion objectives, respectively. The 488 nm ray line of an argon laser was used to detect the GFP fluorescence emission collected in the range between 490 and 540 nm. Three plants per line per time point were observed. **(A,C)** A17 line; **(B,D)** F83005.5 line. **(A,B)** 2 h after inoculation, conidia (sp) are observed in the xylem vessels of both lines. **(C,D)** 24 h after inoculation, conidia are germinating in the xylem vessel of both lines. Co, cortex; hy, hypha; sp, conidia; xy, xylem elements.

During initial stages of root colonization, no difference between the two genotypes was observed (Figure 1). Conidia were sucked into the xylem vessels at the cut root ends by the transpiration stream, in resistant and susceptible line, as observed 2 h after inoculation (Figures 1A,B). Germination of conidia occurred in both lines, within 24 h (Figures 1C,D). Hyphae developed afterwards and colonized the vessels in both lines similarly (data not shown), as already described for the susceptible line F83005.5 (Ben et al., 2013).

**Figure 2:**
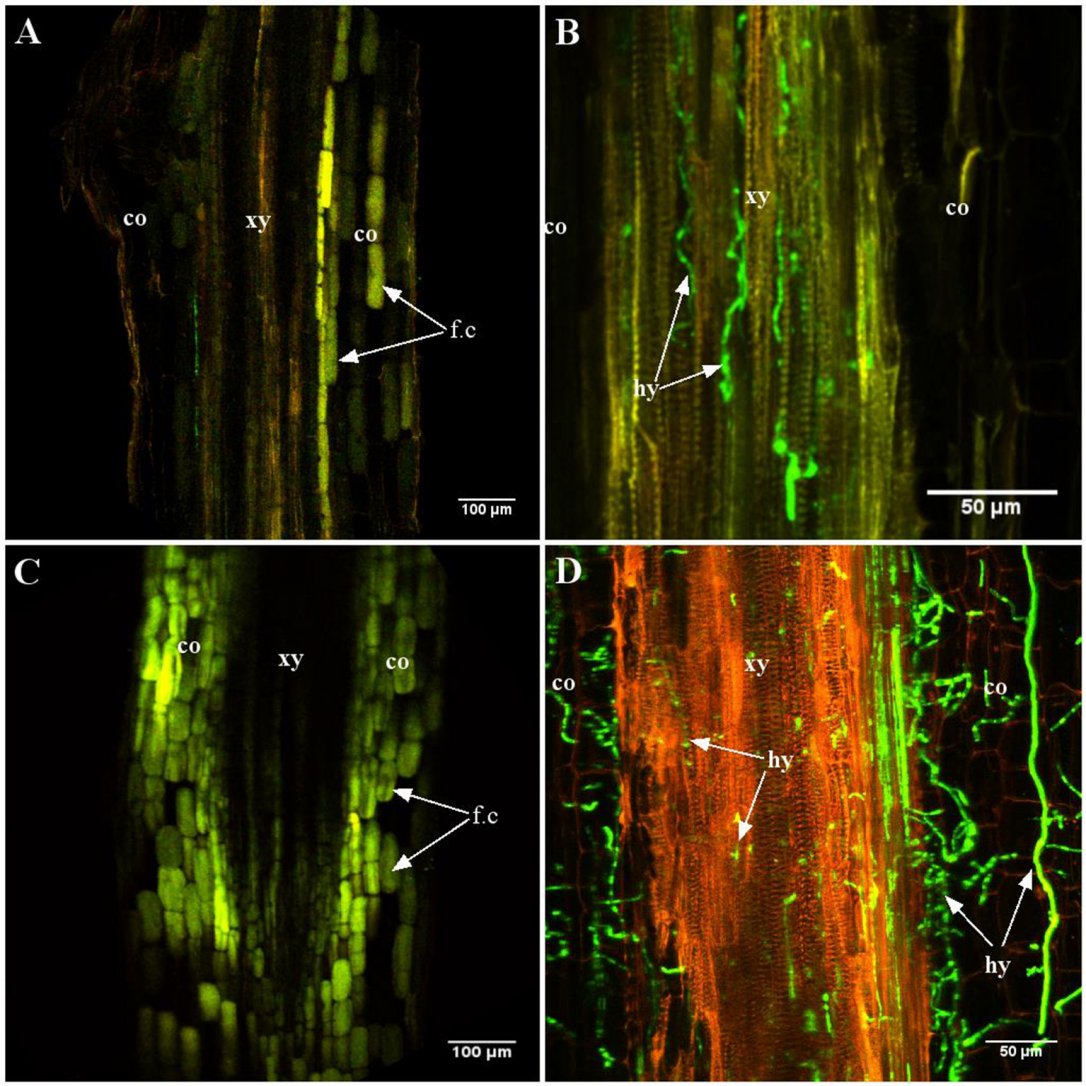
**Late stages of root colonization of A17 (resistant) and F83005.5 (susceptible) by a GFP-expressing strain of *Va* V31-2**. Observations were made on 45 μm thick longitudinal root sections embedded in 5% agarose. Confocal images were acquired with a spectral confocal laser scanning system (SP2 SE, Leica, Germany) equipped with an upright microscope (DM 6000, Leica, Germany). Observations were made using 10 × (HC PL Fluotar, N.A. 0.3) and 40 × (HCX PL APO, N.A. 0.8) dry and water immersion objectives, respectively. The 488 nm ray line of an argon laser was used to detect the GFP fluorescence emission collected in the range between 490 and 540 nm. Three plants per line per time point were observed. **(A,C)** line A17; **(B,D)** line F83005.5. **(A,B)** 7 days after inoculation, the presence of the pathogen is not observed in the resistant line **(A)** while hyphae are observed in the xylem vessels of the susceptible line **(B)**. **(C,D)** 12 days after inoculation, the pathogen is observed only in the susceptible line **(D)**. In roots of the resistant line, numerous cells contain auto-fluorescent compounds **(C)**. co, cortex; hy, hypha; xy, xylem elements; fc, soluble fluorescent compounds.

Differences became apparent at intermediate stages [4–7 days post-inoculation (dpi)] (Figure 2). Indeed, at 7 dpi, A17 roots were devoid of *Va* (Figure 2A) while in roots of the susceptible line, hyphae were abundant in the central cylinder (Figure 2B). Typical for vascular diseases, colonization at this stage was strictly limited to the xylem vessels. At latest stages, the roots of susceptible plants were highly colonized and hyphae were also growing in the cortex (Figure 2D). In contrast, cortical cells of the resistant line showed intensive auto-fluorescence suggesting the accumulation of soluble phenolic compounds (Figures 2A,C; Supplementary Figure S2). Staining with the chitin-binding lectin WGA-FITC did not reveal any hyphae in roots of line A17 at 10 dpi, demonstrating that absence of GFP fluorescence was not due to metabolic inactivity of *Va* (Supplementary Figure S3).

The disappearance of *Va* in line A17 as observed by microscopy, was confirmed by quantification of fungal DNA in *M. truncatula* roots and aerial parts by qPCR using *Va*-specific primers (Supplementary Figure S4). It can be concluded that the fungus was eliminated from roots of the resistant line A17 plants after initial colonization of the vessels.

### *V. alfalfae* strain V31-2 does not contain avirulence gene *Ave1*

Strain V31-2, together with strains *V. albo-atrum* LPP0323 and *V. dahliae* JR2 used in previous studies (Negahi et al., 2013), was characterized by PCR with species-specific primers (Inderbitzin et al., 2013; Supplementary Figure S5). The results confirmed that the alfalfa isolate V31-2 belongs to the newly formed species *V. alfalfae*, and incidentally showed that strain LPP0323 belongs to *V. nonalfalfae*. PCR with *Ave1*-specific primers (de Jonge et al., 2012) conducted on these 3 strains showed that neither V31-2 nor LPP0323 contained an *Ave1* homolog, in contrast to the positive control *Vd* JR2. In *Ve1* tomato, resistance to race 1 *V. dahliae* and *V. albo-atrum* is mediated through recognition of the fungal *Ave1* gene. The absence of *Ave*1 in V31-2 suggests that resistance in *M. truncatula* toward this strain is different from the *Ve1*-*Ave*1 interaction described in tomato.

### Resistant and susceptible lines exhibit specific transcriptional reprogramming early after inoculation with *V. alfalfae*

To get more insight into resistance mechanisms which eliminate *Va* from roots in line A17, a transcriptomic study was undertaken. Based on root colonization pattern, the period between 0 and 24 hpi was considered as decisive, preceding the accumulation of putative defense compounds, and elimination of the pathogen. Two independent experiments with lines A17 and F83005.5 (mock-inoculated, *Va*-inoculated) exhibiting similar time course of infection were conducted (Supplementary Figure S6). cDNA libraries prepared from RNA extracted from roots at t0 (before inoculation) and from roots at 4, 8, and 24 hpi pooled thereafter (named “Early” pools) were constructed. Between 13 and 29 million reads were obtained for each of the 12 libraries by MACE sequencing (Supplementary Table S2).

Sequence mapping on the *M. truncatula* genome gave a total number of 58,186 putative genes. This was reduced to less than half when only genes with ≥5 reads in at least two libraries were considered, with 17,978 sequences for A17 and 18,089 for F83005.5. Among them, 1261 genes were differentially expressed between the two lines at t0, with 735 (58%) over-expressed in A17, establishing a strong genotype effect on basal gene expression (Supplementary Table S3). Interestingly, analysis of GO term enrichment showed that functions associated to “response to stimulus” and to secondary metabolism were strongly enriched for the transcripts with higher expression level in resistant line A17. More strikingly, the category “defense response” was only found in this set of differentially expressed genes (DEGs; Table 1). Line F83005.5 exhibited higher expression of genes involved in primary metabolism, and in categories “biological regulation and signaling,” “localization,” “cellular component organization and process,” and “developmental process.” These strong and large differences in gene expression before inoculation suggest that the resistant line might possess preformed defenses or might be better prepared to induce a fast and efficient response.

**Table 1:** **Enriched Gene Ontology (GO) categories of differentially expressed genes in *M. truncatula* lines A17 and F83005.5 before *Verticillium* inoculation (i.e., at t0)**.

**GO ID**	**GO term definition**	**O**	**U**	**FDR O**	**FDR U**
**METABOLIC PROCESS**					
GO:0006006	Glucose metabolic process			ns	0.00016
GO:0006081	Cellular aldehyde metabolic process			ns	0.0018
GO:0044275	Cellular carbohydrate catabolic process			ns	0.0076
GO:0042398	Cellular amino acid derivative biosynthetic process			2.7e-13	0.00075
GO:0000097	Sulfur amino acid biosynthetic process			ns	0.00013
GO:0009065	Glutamine family amino acid catabolic process			ns	0.00031
GO:0009084	Glutamine family amino acid biosynthetic process			ns	0.0071
GO:0009081	Branched chain family amino acid metabolic process			ns	0.0079
GO:0009095	Aromatic amino acid family biosynthetic process, prephenate pathway			0.0048	ns
GO:0032787	Monocarboxylic acid metabolic process			2e-06	1.8e-12
GO:0046395	Carboxylic acid catabolic process			0.00061	0.0017
GO:0006099	Tricarboxylic acid cycle			ns	0.0036
GO:0006631	Fatty acid metabolic process			ns	0.0021
GO:0006184	GTP catabolic process			ns	0.009
GO:0019748	Secondary metabolic process			9.4e-18	2.7e-05
GO:0019438	Aromatic compound biosynthetic process			4.7e-21	0.00026
GO:0018130	Heterocycle biosynthetic process			ns	0.0027
GO:0055114	oxidation reduction			7.2e-05	ns
GO:0006032	Chitin catabolic process			0.009	ns
GO:0046165	Alcohol biosynthetic process			ns	5.8e-06
GO:0009108	Coenzyme biosynthetic process			ns	0.0056
**RESPONSE TO STIMULUS**					
GO:0009607	Response to biotic stimulus			1.4e-29	2.4e-06
GO:0006952	Defense response			1.6e-13	ns
GO:0010033	Response to organic substance			7.4e-08	1.1e-23
GO:0009416	Response to light stimulus			7.4e-08	ns
GO:0006979	Response to oxidative stress			2.5e-05	0.0019
GO:0010038	Response to metal ion			0.00014	3.1e-07
GO:0009605	Response to external stimulus			0.00028	ns
GO:0009725	Response to hormone stimulus			ns	1.9e-10
GO:0009415	Response to water			ns	0.0023
GO:0051716	Cellular response to stimulus			ns	0.0036
**BIOLOGICAL REGULATION, SIGNALING**					
GO:0043086	Negative regulation of catalytic activity			0.0011	ns
GO:0048523	Negative regulation of cellular process			ns	0.0036
GO:0044092	Negative regulation of molecular function			0.0011	0.0075
GO:0010557	Positive regulation of macromolecule biosynthetic process			ns	0.00085
GO:0031328	Positive regulation of cellular biosynthetic process			ns	5.8e-06
GO:0032268	Regulation of cellular protein metabolic process			ns	0.0023
GO:0007242	Intracellular signaling cascade			ns	0.0095
GO:0032259	Methylation			0.0041	1.3e-06
GO:0001510	RNA methylation			ns	0.00038
GO:0006412	Translation			ns	5e-09
**LOCALIZATION AND TRANSPORT**					
GO:0006605	Protein targeting			0.00069	1.1e-06
GO:0006839	Mitochondrial transport			ns	0.0025
**DEVELOPMENTAL PROCESS**					
GO:0048869	Cellular developmental process			ns	2.6e-05
GO:0007275	Multicellular organismal development			ns	0.0045
**CELLULAR PROCESS**					
GO:0044085	Cellular component biogenesis			ns	0.00026
GO:0051128	Regulation of cellular component organization			ns	0.0013
GO:0051276	Chromosome organization			ns	0.0065
GO:0034621	Cellular macromolecular complex subunit organization			ns	0.0036
**OTHER**					
GO:0009405	Pathogenesis			0.00094	ns
GO:0022414	Reproductive process			ns	0.0033

*340 (46%) over-expressed and 484 (92%) under-expressed genes in line A17 compared to line F83005.5 at t0 were annotated with GO terms using BLAST2GO, respectively. Over-represented GO terms for biological process (adjusted P = 0.01) were identified using agriGO. To clarify the overview, only the significantly enriched GO terms of deeper level in the hierarchical tree graph are mentioned herein and labeled by their GO ID, term definition and statistical information. The degree of color saturation is positively correlated to the enrichment level of the term. O, Over-expressed genes in A17 compared to F83005.5; U, Under-expressed genes in A17 compared to F83005.5; FDR, FDR under dependency computed with the Yekutieli's multi-test correction method; ns, not significantly enriched*.

**Figure 3:**
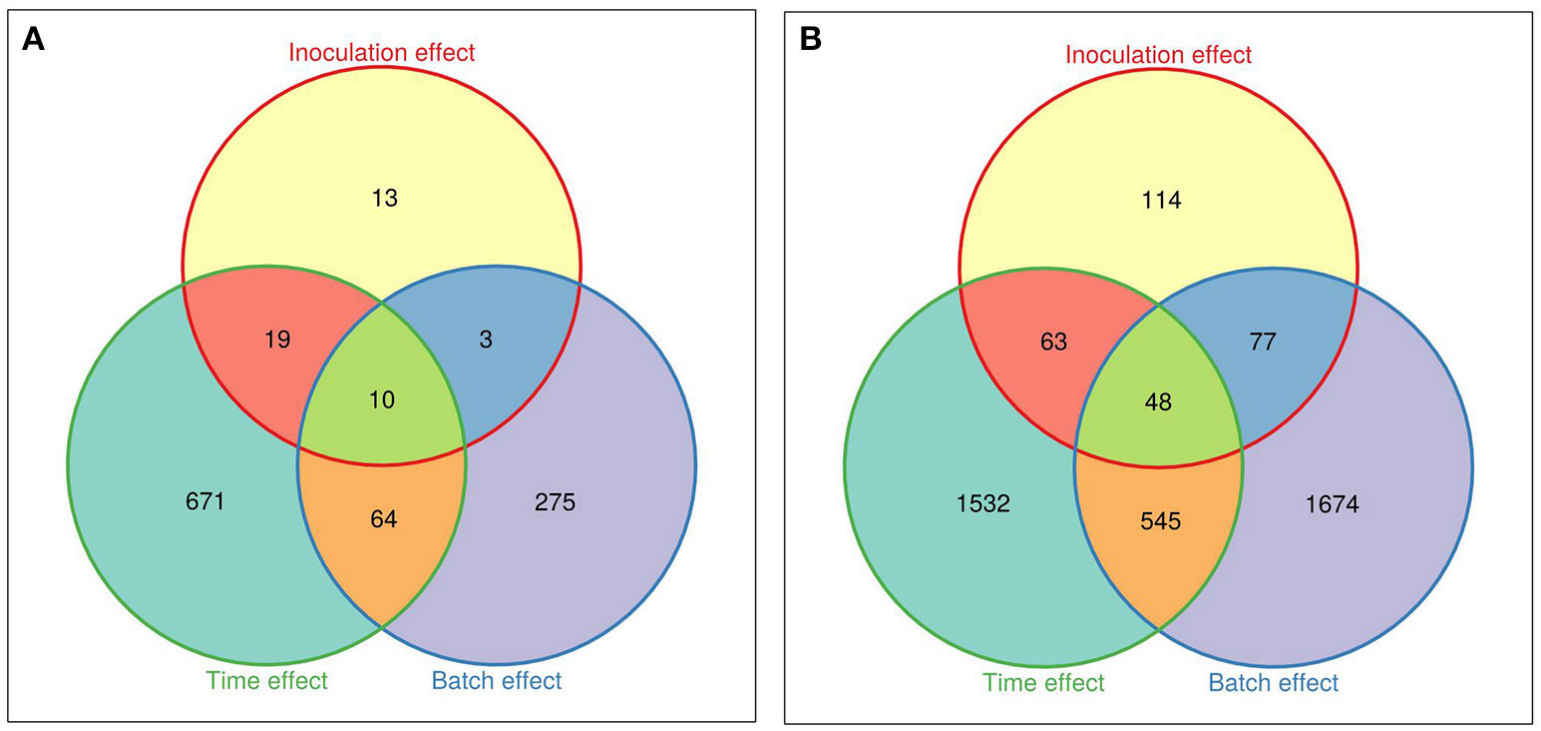
**Venn diagrams of the differentially expressed genes in roots of *M. truncatula* lines. (A)** Differentially expressed genes in the resistant line A17. **(B)** Differentially expressed genes in the susceptible line F83005.5. “Inoculation effect” represents the comparison between *Va*-inoculated and mock-inoculated samples, “Time effect” shows the comparison of mock-inoculated at t0 and in pool early, and “Batch effect” corresponds to a comparison between the two experiments (biological repeats). A gene is differentially expressed if the False Discovery Rate (FDR) is <0.05. Venn diagrams were generated with the Vennerable R package (http://r-forge.r-project.org/projects/vennerable).

Due to the strong genotype effect, independent data analyses were performed for each *M. truncatula* line. Three groups of DEGs were distinguished, corresponding to genes regulated by time/development (Early-Mock vs. t0), by inoculation (Early-inoculated vs. Early-Mock), or by experimental batch factors (Experiment 1 vs. Experiment 2; Figures 3A,B). In total, 1055 and 4053 DEGs were identified in A17 and F83005.5, respectively, evidencing a stronger transcriptional response in the susceptible line, although colonization at this stage was still very low and not much different from that in the resistant line (Figure 1). Among the 45 DEGs responding to inoculation in the resistant line A17 (0.3% of the 17,978 genes), 13 responded specifically to *Va*, 29 responded also to development; most genes (72%) were induced (Supplementary Table S4). Among the 302 DEGs responsive to inoculation in the susceptible line F83005.5 1.7% of the 18,089 genes), 114 (37.7%) responded specifically to *Va* and 111 (36.7%) responded also to development; here, up- and down-regulated genes accounted each for roughly half (45 and 55%) of the DEGs (Supplementary Table S5). The gene expression profiles revealed by MACE were validated by qRT-PCR for 24 DEGs which appeared up- or down-regulated in inoculated A17 and/or F83005.5 (Supplementary Table S6). Gene expression levels were highly similar with both methods (Pearson's correlation coefficient, *r* = 0.86) supporting the reliability of MACE results.

Twenty DEGs showed similar expression patterns in both lines. Nineteen were functionally annotated, including genes involved in secondary metabolism, oxidation-reduction process, response to stress, and regulation of cellular process (Table 2). Both A17 and F83005.5 showed increased expression of three chalcone synthase genes after inoculation with *Va*, but induction was stronger in line A17.

**Table 2:** **Genes responding similarly to inoculation in the resistant (A17) and susceptible (F83005.5) *M. truncatula* lines**.

**ID Mt v3.5**	**ID Mt v4**	**Annotation**	**FC A17**	**FC F83005.5**	**Biological process GO terms**
**UP-REGULATED GENES**					
Contig_64564_1.1	Medtr7g103390.1	Myb sant-like dna-binding domain protein	7,3	4,5	Regulation of cellular process
Medtr7g086320.1	Medtr7g086320.1	Hypothetical protein MTR_7g086320	5,8	5,6	–
Medtr5g022390.1	Medtr5g022380.1	Rhodanese-related sulfurtransferase	4,7	2,7	Transferase activity
Medtr1g097910.1	Medtr1g097910.1	Chalcone synthase	4,3	1,7	Flavonoid biosynthetic process
TC187179	Medtr3g089940.2	Alcohol dehydrogenase	4,2	2,3	Oxidation-reduction process
Contig_240964_1.1	Medtr2g089835.1	Wound-responsive family protein	3,9	4,7	–
Medtr1g098140.1	Medtr1g098140.1	Chalcone synthase	3,7	1,9	Flavonoid biosynthetic process
Medtr1g083950.1	Medtr1g083950.1	Universal stress protein a-like protein	3,6	2,7	–
TC194155	Medtr1g097935.1	Chalcone synthase	3,2	2,0	Flavonoid biosynthetic process
Medtr7g055630.1	Medtr7g055630.1	Ankyrin repeat protein	2,7	1,7	–
Medtr5g022380.1	Medtr5g018720.1	nodulin family protein	2,5	3,0	Response to karrikin
TC196800	Medtr2g016650.1	Hypothetical protein	2,4	2,3	Anaerobic respiration
Contig_7825	–	–	2,4	2,4	–
**DOWN-REGULATED GENES**					
Medtr8g078170.1	Medtr8g078170.1	Coiled-coil domain-containing protein	−2,3	−1,6	–
Medtr8g022300.1	Medtr8g022300.1	Dormancy auxin associated protein	−2,6	−2,5	–
Contig_167649_1.1	Medtr3g088845.1	2-oxoisovalerate dehydrogenase subunit alpha	−2,7	−4,9	Oxidation-reduction process
Medtr2g096120.1	Medtr2g082050.1	Uncharacterized loc101218723	−2,7	−3,6	–
TC176640	Medtr1g083440.1	Dormancy auxin associated protein	−3,8	−3,4	Response to brassinosteroid stimulus
TC191486	Medtr1g029500.1	f-box protein skp2a	−4,1	−4,1	–
Contig_70372_1.1	Medtr8g012795.1	Defensin-like protein	−5,4	−3,5	Response to stress

*ID Mt v3.5 and ID Mt v4 correspond, respectively to gene IDs in versions v3.5 and v4 of M. truncatula genome. FC A17 (respectively, FC F83005.5) values correspond to log2 of the ratio [mean of normalized counts in Va-inoculated condition/ mean of normalized counts in control condition]; Va, Verticillium alfalfae*.

### *In silico* functional analysis indicates induction of a targeted defense response in the resistant line

To get further insights into the biological pathways involved in the response to *Va* infection, GO term enrichment analyses were performed for the DEGs responding to fungal inoculation in each line. Regarding line A17, all significantly enriched functional categories contained only up-regulated DEGs and concerned a limited number of biological processes (Table 3). The category “response to stimulus” included notably “response to biotic stimulus,” “defense response,” and “response to organic substance,” and the category “metabolic process” included “aromatic compound,” “amino acid derivative,” and “secondary metabolism.” This picture suggests a targeted and organized biological response of the resistant line early after pathogen inoculation. In contrast, the response to *Va* in the susceptible line F83005.5 appeared more complex and disordered. Processes related to “response to stimulus” as well as “metabolism process” including very diverse biological pathways were highly affected (Table 4). Among those, single functional categories such as “cellular catabolic process” or “response to biotic stimulus” were both significantly induced and repressed. A majority of cellular pathways such as “response to oxidative stress” and “response to hormone stimuli” appeared to be down-regulated, and all genes of the category “biological regulation,” among them notably “ion homeostasis” were consistently repressed. Strikingly, the GO term associated with “defense response” was not significantly enriched in the susceptible line.

**Table 3:** **Comparison of enriched GO terms in up- and down-regulated genes in the resistant line A17 in response to *V. alfalfae* inoculation**.

**GO ID**	**GO term definition**	**U**	**D**	**FDR U**	**FDR D**
**METABOLIC PROCESS**					
GO:0019438	Aromatic compound biosynthetic process			0.0012	ns
GO:0042398	Cellular amino acid derivative biosynthetic process			0.0012	ns
GO:0019748	Secondary metabolic process			0.0012	ns
**RESPONSE TO STIMULUS**					
GO:0009607	Response to biotic stimulus			0.0012	ns
GO:0006952	Defense response			0.025	ns
GO:0010033	Response to organic substance			0.027	ns

*20 (61%) up-regulated and 6 (50%) down-regulated genes in response to V. alfalfae in line A17 compared to control condition were annotated with GO terms using BLAST2GO, respectively. Over-represented GO terms for biological process (adjusted P = 0.05) were identified using agriGO. To clarify the overview, only the significantly enriched GO terms of deeper level in the hierarchical tree graph are mentioned herein and labeled by their GO ID, term definition and statistical information. The degree of color saturation is positively correlated to the enrichment level of the term. U, Up-regulated genes in Va-inoculated condition compared to control; D, Down-regulated genes in Va-inoculated condition compared to control; FDR, FDR under dependency computed with the Yekutieli's multi-test correction method; ns, not significantly enriched; Va, Verticillium alfalfae*.

**Table 4:** **Comparison of enriched GO terms in up- and down-regulated genes in the susceptible line F83005.5 in response to *V. alfalfae* inoculation**.

**GO ID**	**GO term definition**	**U**	**D**	**FDR U**	**FDR D**
**METABOLIC PROCESS**					
GO:0005985	Sucrose metabolic process			0.003	ns
GO:0006012	Galactose metabolic process			ns	0.022
GO:0019319	Hexose biosynthetic process			ns	0.022
GO:0006081	Cellular aldehyde metabolic process			0.047	0.003
GO:0006090	Pyruvate metabolic process			ns	0.022
GO:0042398	Cellular amino acid derivative biosynthetic process			1.2e-09	ns
GO:0009063	Cellular amino acid catabolic process			ns	0.0041
GO:0006551	Leucine metabolic process			ns	0.0094
GO:0006558	L-phenylalanine metabolic process			0.016	ns
GO:0008654	Phospholipid biosynthetic process			ns	0.024
GO:0009247	Glycolipid biosynthetic process			ns	0.027
GO:0019748	Secondary metabolic process			9.5e-11	ns
GO:0019438	Aromatic compound biosynthetic process			2.2e-09	ns
GO:0032787	Monocarboxylic acid metabolic process			0.0085	ns
GO:0046700	Heterocycle catabolic process			ns	0.013
GO:0016117	Carotenoid biosynthetic process			ns	0.032
GO:0006800	Oxygen and reactive oxygen species metabolic process			4.4e-06	ns
GO:0015979	Photosynthesis			ns	0.036
GO:0015994	Chlorophyll metabolic process			ns	0.00009
GO:0044248	Cellular catabolic process			0.0087	0.00018
GO:0051187	Cofactor catabolic process			ns	0.0012
**RESPONSE TO STIMULUS**					
GO:0010033	Response to organic substance			0.00004	3.1e-21
GO:0009607	Response to biotic stimulus			4.9e-05	0.00027
GO:0009719	Response to endogenous stimulus			0.017	5.7e-07
GO:0009725	Response to hormone stimulus			ns	5.7e-07
GO:0010035	Response to inorganic substance			ns	0.0041
GO:0006979	Response to oxidative stress			ns	0.029
GO:0009267	Cellular response to starvation			ns	0.022
GO:0009415	Response to water			ns	0.019
**BIOLOGICAL REGULATION, SIGNALING, AND LOCALIZATION**					
GO:0006826	Iron ion transport			ns	0.00071
GO:0006879	Cellular iron ion homeostasis			ns	0.00092
GO:0048519	Negative regulation of biological process			0.00065	0.0012
GO:0045892	Negative regulation of transcription DNA-dependent			ns	0.019
GO:0023033	Signaling pathway			ns	0.02

*70 (52%) up-regulated and 112 (67%) down-regulated genes in response to V. alfalfae in F83005.5 line compared to control condition were annotated with GO terms using BLAST2GO, respectively. Over-represented GO terms for biological process (adjusted P = 0.05) were identified using agriGO. To clarify the overview, only the significantly enriched GO terms of deeper level in the hierarchical tree graph are mentioned herein and labeled by their GO ID, term definition, and statistical information. The degree of color saturation is positively correlated to the enrichment level of the term. U, Up-regulated genes in Va-inoculated condition compared to control; D, Down-regulated genes in Va-inoculated condition compared to control; FDR, FDR under dependency computed with the Yekutieli's multi-test correction method; ns, not significantly enriched; Va, Verticillium alfalfae*.

To get a more focused vision into specific cellular processes, we overlaid DEGs responding to *Va* inoculation onto metabolic networks related to biotic stress response using MapMan (Usadel et al., 2005; Supplementary Figure S7). The response of the resistant line A17 was characterized by induction of a few metabolic pathways putatively involved in pathogenic interactions. In particular “plant secondary metabolism” accounted for eight of the significantly induced genes in A17 (Supplementary Figure S7A), with genes involved in metabolism of lignins and lignans, phenylpropanoids, and flavonoids (including chalcones and isoflavonoids; Supplementary Figure S8A). Genes involved in secondary metabolic process were induced in the susceptible line as well, but with lower expression levels (Supplementary Figure S8B). In addition, many cellular functions were modified (induced and/or repressed) in line F83005.5 in response to *Verticillium*, including genes participating to cell wall and proteolysis, to defense (mostly PR proteins), to oxidative stress, signaling, and transcription factors. Notably, expression of genes involved in phytohormone signaling was strongly affected in this line: genes related to ethylene (ET), jasmonate (JA), abscisic acid (ABA), and auxin signaling were induced whereas genes of salicylic acid (SA) signaling were repressed (Supplementary Figure S7B).

Experiments where plants were pre-treated with ET, MeJA, ABA, auxin, and SA showed that ABA, SA, and auxin protected against symptoms and fungal colonization and ET delayed disease symptoms whereas MeJA had no effect (Supplementary Figures S9, S10). None of these hormones had a direct effect on the fungus (Supplementary Table S7). Hence, the induction of these genes in line F83005.5 does not seem sufficient to fully trigger defenses regulated by the respective hormone signaling pathways.

Incidentally “Response to abiotic stress” showed a similar contrasted pattern in the two lines: induction of genes in A17 and simultaneous induction and repression of genes in F83005.5.

On the whole, GO enrichment and MapMan analyses suggest a structured molecular response in the resistant line A17 leading to the onset of defense mechanisms early after *Va* inoculation. In contrast, the susceptible line F83005.5 suffered a complete transcriptional dysregulation of plant metabolism and defense pathways which probably favors disease development.

### Gene co-expression network analysis identifies five functional modules and transcriptional factors underlying resistance against *V. alfalfae*

**Figure 4:**
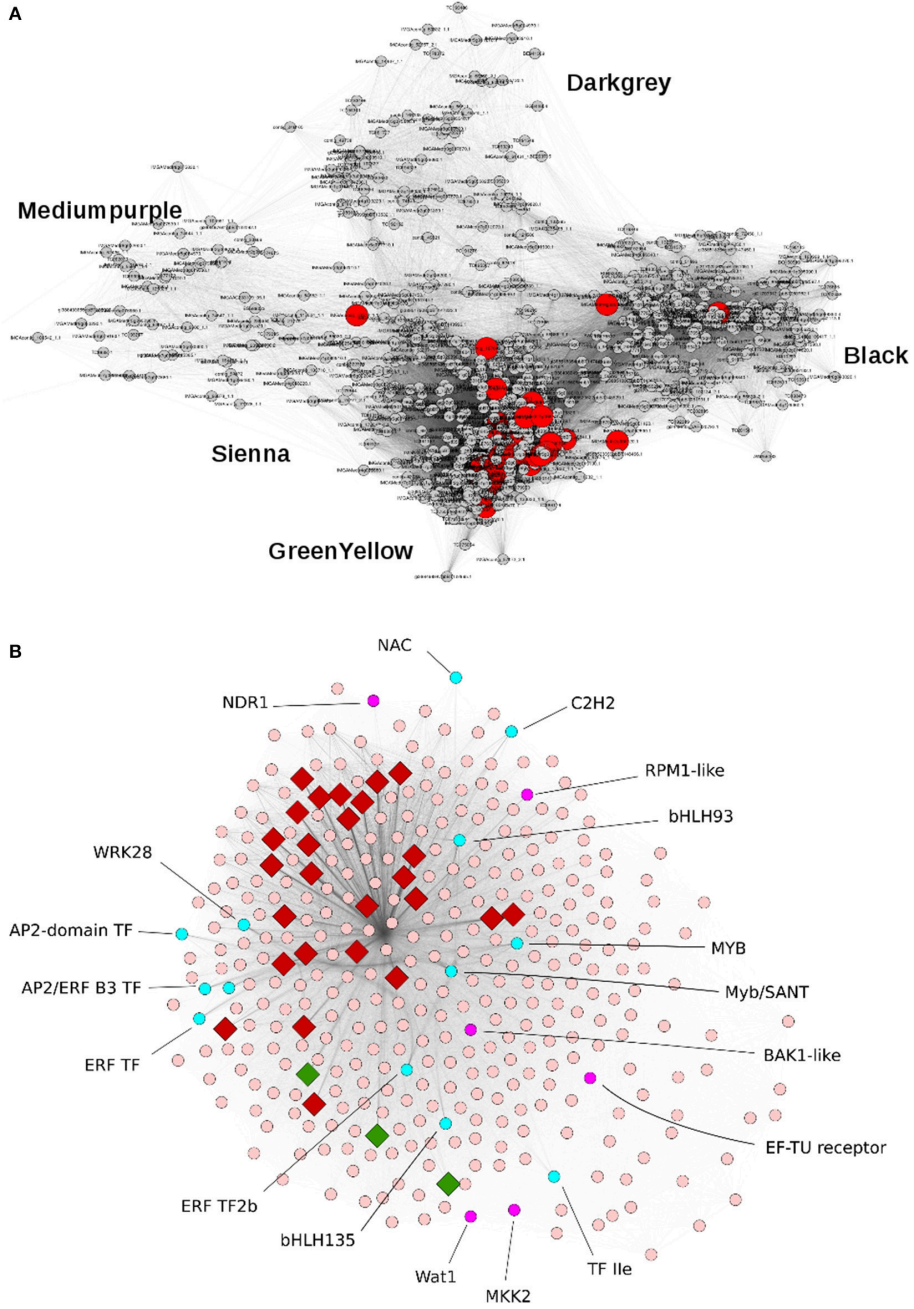
**Principal modules of the gene co-expression network associated to inoculation response in the resistant *M. truncatula* line A17. (A)** Representation of the Weighted Gene Co-Expression Network of 794 genes in the resistant line A17. Five modules can be distinguished and are named by color codes. Module Greenyellow contains 369 genes, Black 243 genes, Darkgray 82 genes, Mediumpurple 65 genes, and Sienna 35 genes. Genes differentially expressed after *Va* V31-2 inoculation are shown in red. **(B)** Representation of co-expression module Greenyellow which contains 369 genes. A rhombus indicates that the gene is differentially expressed after *Va* V31-2 inoculation. In red: induced genes, in green: repressed genes, in cyan: transcription factors.

We further investigated the transcriptome organization in resistant line A17 by applying weighted gene co-expression network analysis (WGCNA) to 2083 sequences exhibiting a coefficient of variation of at least 0.35 across the six libraries (t0, Early-Mock, Early-inoculated, two repetitions). This dataset comprised all 45 inoculation-responsive DEGs identified above. Complementary to the analyses described above which considered only DEGs with a FDR of <0.05, this approach will further reveal subtle effects and show the degree of relationship between genes expression patterns by nodes distance in the graphical presentation. We identified five modules associated to inoculation response which gathered 794 genes (35 to 369 genes per module; Figure 4A, Table 5). This structure points out a coordinated transcriptional response in the incompatible interaction between *M. truncatula* and *V. alfalfae*. In contrast, WGCNA on data from line F83005.5 provided only a blurred picture without any distinct co-expression module, suggesting the absence of a structured transcriptional regulation in the susceptible line after inoculation (data not shown).

**Table 5:** **GO term functional enrichment analysis of the five WGCNA regulatory modules associated with *M. truncatula* root response to *V. alfalfae* inoculation**.

**Module**	**Number of transcripts**	**GO ID**	**GO term definition**		**FDR**
Greenyellow	369	**Metabolism**			
		GO:0005985	Sucrose metabolic process		9.6e-06
		GO:0000272	Polysaccharide catabolic process		4.2e-05
		GO:0006096	Glycolysis		0.00057
		GO:0042398	Cellular amino acid derivative biosynthetic process		3.3e-19
		GO:0009081	Branched chain family amino acid metabolic process		3.5e-08
		GO:0009063	Cellular amino acid catabolic process		0.0001
		GO:0006555	Methionine metabolic process		0.00019
		GO:0032787	Monocarboxylic acid metabolic process		0.0071
		GO:0019748	Secondary metabolic process		4.3e-23
		GO:0019438	Aromatic compound biosynthetic process		4e-17
		GO:0055114	Oxidation reduction		8.2e-14
		**Response to Stimulus**			
		GO:0009607	Response to biotic stimulus		7.1e-25
		GO:0006952	Defense response		2.7e-07
		GO:0009725	Response to hormone stimulus		9.3e-09
		GO:0009416	Response to light stimulus		2e-06
		GO:0010038	Response to metal ion		5e-06
		GO:0051716	Cellular response to stimulus		1.7e-05
		**Biological Regulation, Signaling**			
		GO:0032259	Methylation		1.5e-07
		GO:0065008	Regulation of biological quality		2e-06
		GO:0043086	Negative regulation of catalytic activity		0.0041
		GO:0048523	Negative regulation of cellular process		0.0042
		GO:0007242	Intracellular signaling cascade		0.0035
		**Localization and Transport**			
		GO:0006605	Protein targeting		0.00024
		GO:0008643	Carbohydrate transport		0.00019
		GO:0015671	Oxygen transport		0.0017
		GO:0006865	Amino acid transport		0.0031
		**Developmental, Cellular, and Reproductive Process**			
		GO:0007275	Multicellular organismal development		7.8e-06
		GO:0051704	Multi-organism process		5.2e-05
		GO:0071554	Cell wall organization or biogenesis		0.0039
		GO:0032989	Cellular component morphogenesis		0.0093
		GO:0000003	Reproduction		0.0028
Black	243	**Metabolism**			
		GO:0006730	One-carbon metabolic process		0.004
		GO:0044275	Cellular carbohydrate catabolic process		0.0051
		GO:0006066	Alcohol metabolic process		0.0026
		GO:0022904	Respiratory electron transport chain		0.0061
		**Response to Stimulus**			
		GO:0006950	Response to stress		1.8e-06
		GO:0010033	Response to organic substance		1.5e-05
		**Biological Regulation, Signaling**			
		GO:0048523	Negative regulation of cellular process		0.0003
		**Localization and Transport**			
		GO:0006810	Transport		0.0051
		**Developmental, Cellular and Reproductive Process**			
		GO:0016043	Cellular component organization		0.0026
Darkgray	82	**Metabolism**			
		GO:0009207	Purine ribonucleoside triphosphate catabolic process		7.4e-05
		GO:0009231	Riboflavin biosynthetic process		0.00041
		GO:0019748	Secondary metabolic process		0.0082
		GO:0019438	Aromatic compound biosynthetic process		0.0095
		GO:0046034	ATP metabolic process		0.0043
		**Response to Stimulus**			
		GO:0010035	Response to inorganic substance		0.0008
		**Localization and Transport**			
		GO:0006812	Cation transport		0.002
Mediumpurple	65	**Metabolism**			
		GO:0005975	Carbohydrate metabolic process		0.0013
		GO:0015979	Photosynthesis		0.0074
		GO:0019344	Cysteine biosynthetic process		0.0074
		GO:0006364	rRNA processing		0.0074
		**Response to Stimulus**			
		GO:0009416	Response to light stimulus		3.3e-05
		GO:0006950	Response to stress		0.0059
		**Biological Regulation, Signaling**			
		GO:0048519	negative regulation of biological process		0.00084
		**Developmental, Cellular, and Reproductive Process**			
		GO:0061024	Membrane organization		0.0059
		**Other**			
		GO:0009405	Pathogenesis		0.0072
Sienna	35	**Metabolism**			
		GO:0006081	Cellular aldehyde metabolic process		0.0064
		GO:0006090	Pyruvate metabolic process		0.0045
		GO:0015995	Chlorophyll biosynthetic process		0.0059
		GO:0006546	Glycine catabolic process		0.0045
		GO:0019344	Cysteine biosynthetic process		0.0045
		GO:0009117	Nucleotide metabolic process		0.0063
		GO:0019438	Aromatic compound biosynthetic process		0.0045
		GO:0016117	Carotenoid biosynthetic process		0.0059
		GO:0006733	Oxidoreduction coenzyme metabolic process		0.0045
		GO:0006364	rRNA processing		0.0045
		**Response to Stimulus**			
		GO:0009416	Response to light stimulus		0.0045
		GO:0010033	Response to organic substance		0.0045
		GO:0010035	Response to inorganic substance		0.0082
		**Biological Regulation, Signaling**			
		GO:0045893	Positive regulation of transcription, DNA-dependent		0.0045
		**Localization and Transport**			
		GO:0015931	Nucleobase, nucleoside, nucleotide, and nucleic acid transport		0.0045

*221 (60%) transcripts from Greenyellow module, 146 (60%) from Black module, 55 (68%) from Darkgray module, 48 (74%) from Mediumpurple module, and 28 (80%) from Sienna module were annotated with GO terms using BLAST2GO, respectively. Over-represented GO terms for biological process (adjusted P ≤ 0.01) were identified using agriGO. To clarify the overview, only the significantly enriched GO terms of deeper level in the hierarchical tree graph are mentioned herein and labeled by their GO ID, term definition and statistical information. The degree of color saturation is positively correlated to the enrichment level of the term. FDR: FDR under dependency computed with the Yekutieli's multi-test correction method; ns: not significantly enriched; WGCNA: Weighted Gene Co-expression Network Analysis*.

Statistical analysis of GO term enrichment revealed distinct functional assignments for the different co-expression modules (Table 5). Biological processes involved in response to various biotic and abiotic stimuli were significantly over-represented in all modules, and three of them (Greenyellow, Black, and Darkgray) appeared to be related to specific regulatory pathways such as “negative regulation of biological process” or to different metabolic activities, with some involved in defense against pathogens (e.g., “secondary metabolic process”).

The Greenyellow module is highly enriched in biological functions related to plant defense and encompasses genes exhibiting expression profiles the most correlated to inoculation response. Notably, it contains 29 (64%) of the previously identified DEGs responding to *Va* inoculation in line A17, together with non-differentially expressed genes (Figure 4B). Hence it appeared to be of major importance for resistance against *Va*. Detailed examination of this resistance-associated module led to the identification of genes known to play key roles in plant-pathogen interactions, such as genes involved in plant defense (e.g., encoding chitinases, disease resistance RPM1-like proteins, pathogenesis-related protein PR10 or PR5-like receptor kinase (PRK5), LRR protein, …), in ROS metabolism (e.g., peroxidases…), and in secondary metabolism (e.g., PAL, chalcone synthases,…; Supplementary Table S8). Phytohormone-responsive genes, most of them related to ET, ABA, and auxin signaling, are also present in this module, together with genes encoding receptor-like kinases and protein kinases and 21 putative transcription factors (TFs) from different gene families (namely, AP2/ERF and B3, ethylene responsive, MYB and MYB/SANT, NAC, bHLH, WRKY, TIFY, LOB, Trihelix family protein, and C2H2-type zinc finger protein families). Homologs of several genes involved in PAMP-triggered immunity (PTI) were identified, such as BAK1 (FM886833.1), MKK2 (Medtr1g113960.1), EF-Tu Receptor (EFR, Medtr5g026090.1), NDR1 (contig_99115_1.1), and Wat1 (walls are thin 1; Medtr3g072500). A KEGG analysis revealed the presence of additional homologs of genes related to PTI, namely CNGC10 (cyclic nucleotide gated channel 10), HSP90 (heat shock protein 90), and CERK1 (chitin elicitor receptor kinase 1). These genes may play a crucial role in the perception of *Va* and in signal transduction in *M. truncatula* roots as well as in the subsequent transcriptional regulation leading to resistance.

**Figure 5:**
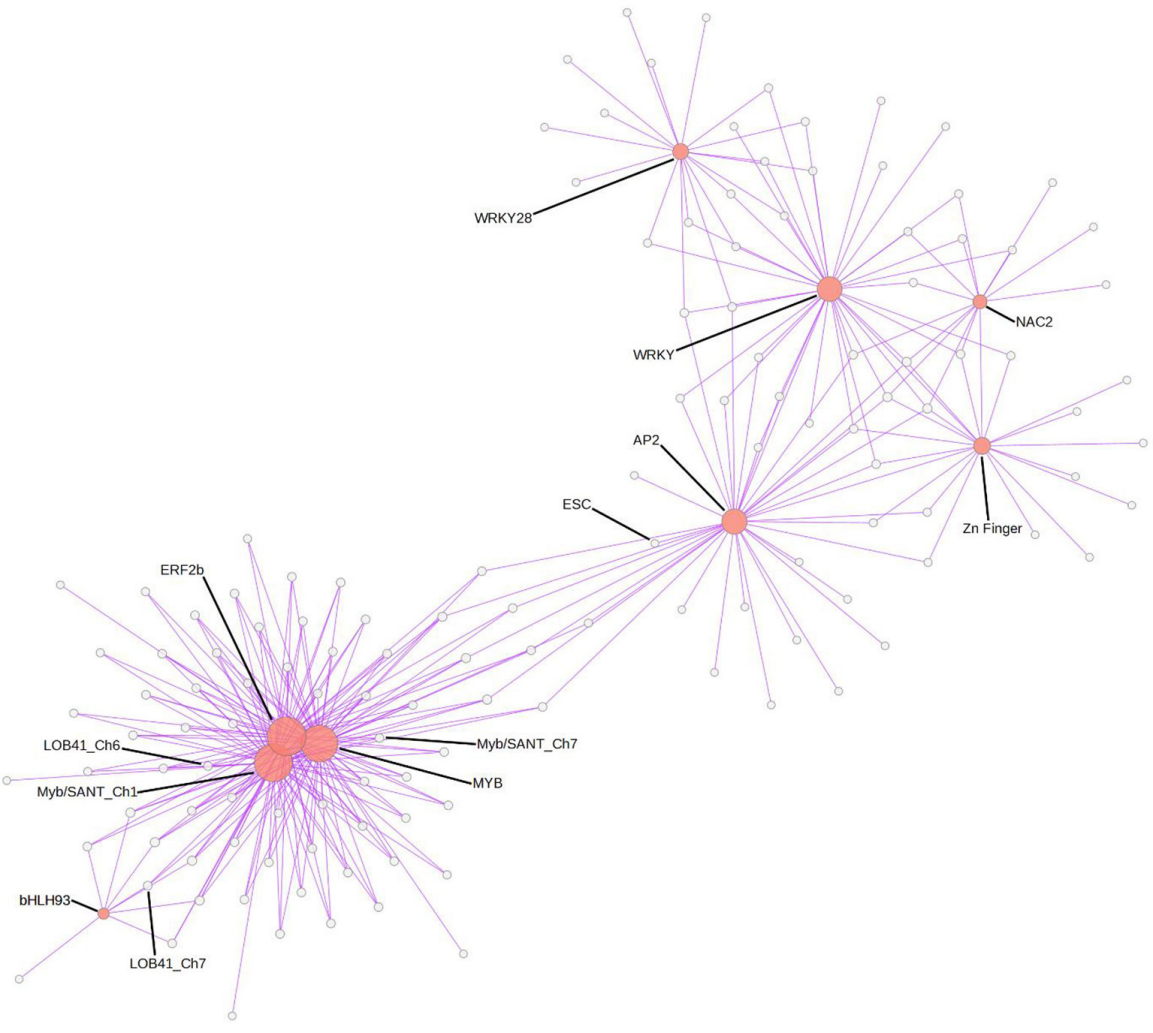
**Gene co-expression network in response to various root pathogens predicted using LegumeGRN**. Affymetrix probes of the Greenyellow module genes were identified by BLAST Search on *Medicago truncatula* Gene Expression Atlas. At least one probe was identified for 333 (/369) genes. A predicted co-expression network for 186 genes (http://legumegrn.noble.org/) using a stringent Pearson correlation threshold (Pearson correlation coefficient >±0.8) was identified according to the *Medicago truncatula* Gene Expression Atlas expression data of A17 roots inoculated with *Aphanomyces euteiches, Ralstonia solanacearum, Phymatotrichopsis omnivore*, and *Macrophomina phaseolina*. AP2 (AP2 domain class transcription factor, Mtr.42129.1.S1_at), bHLH93 (Transcription factor bHLH93; Mtr.44005.1.S1_at), ERF2b (Ethylene responsive transcription factor 2b; Mtr.43313.1.S1_at), ESC (DNA-binding protein escarola-like; Mtr.41053.1.S1_at), LOB41_Ch6 and LOB41_Ch7 (LOB domain-containing protein 41-like; Mtr.50046.1.S1_at and Mtr.40344.1.S1_at, respectively), MYB (MYB-like transcription factor family protein; Mtr.22332.1.S1_at), Myb/SANT_Ch1 and Myb/SANT_Ch7 (Myb/SANT-like DNA-binding domain protein; Mtr.32196.1.S1_at and Mtr.11595.1.S1_at, respectively); NAC2 (NAC domain-containing protein 2-like; Mtr.37370.1.S1_at), WRKY (WRKY family transcription factor; Mtr.44416.1.S1_at) and WRKY28 (probable WRKY transcription factor 28-like; Mtr.38202.1.S1_at), Zn Finger (zinc finger protein zat11-like; Mtr.41238.1.S1_at).

Finally, to address the question if the Greenyellow co-regulatory module was specific for *M. truncatula* resistance toward *Verticillium* or potentially involved in the response to other root pathogens, we used the LegumeGRN prediction server (Wang M. et al., 2013). An *in silico* analysis of co-expression patterns of the 333 (90%) genes from the Greenyellow module which possess Affymetrix probes on transcriptional data of *M. truncatula* roots inoculated with *Aphanomyces euteiches, Macrophomina phaseolina, Phymatotrichopsis omnivore*, and *Ralstonia solanacearum* led us to identify a stringent co-expression network gathering 186 genes (Figure 5). This highlights a substantial level of conservation in the transcriptional response of *M. truncatula* toward various root pathogens. Indeed, a high proportion (56%) of the genes with Affymetrix probes from the Greenyellow module are also co-regulated in response to other root pathogens (either fungi, bacteria, or oomycetes). A core of 15 TFs holds central positions within this co-regulatory module. Hence, not only defense mechanisms but also important regulators of gene expression are conserved among the responses of *M. truncatula* to different root pathogens.

## Discussion

### An undescribed root resistance against *Verticillium* wilt in *M. truncatula*

The response against *V. alfalfae* in resistant *M. truncatula* is different from the well-characterized *Ve*1 dependent resistance against *V. dahliae* and *V. albo-atrum* in host plants such as tomato, cotton, or *Solanum torvum* (Fradin et al., 2009; Liu et al., 2012; Zhang et al., 2012). Homologs of *Ve*1 were detected in the *M. truncatula* genome but were not localized under the resistance QTLs against *Va* V31-2 (Ben et al., 2013) and are not expressed at important early stages of the interaction, since they were not detected in our MACE libraries. Ve1 recognizes Ave1, the *Verticillium* effector of race1 strains (Fradin et al., 2011). *Ave*1 homologs were found in three fungal and many plant species; the *Fusarium* and *Cercospora* homologs were both recognized by tomato *Ve*1, supporting the hypothesis that *Ve*1—*Ave*1 mediated resistance had traits of PAMP-triggered immunity (de Jonge et al., 2012). *Va* strain V31-2 does not possess *Ave*1 homologs although they were reported in the alfalfa strain VaMS102 (de Jonge et al., 2012).

The study of root colonization showed that after initial colonization of the stele, the fungus was eliminated from roots of the resistant line A17. In other plants, resistance takes place in the shoot. *V. dahliae* and *V. longisporum* were shown to infect roots of resistant and susceptible hosts but resistant hosts inhibited pathogen development in the shoot (Heinz et al., 1998; Eynck et al., 2009; Zhang W. W. et al., 2013). The destruction of the fungus by the plant, and the absence of both *Ave*1 in *Va* strain V31-2 and of *Ve*1 in line A17 suggest that resistance to *Verticillium* wilt in *M. truncatula* A17 is novel and different from that described in other plants.

### The transcriptional response of susceptible and resistant lines to *Verticillium* involves some shared genes, but with different expression levels

A common transcriptional response involving a few genes related to flavonoid biosynthesis and stress response was found in both *M. truncatula* lines. However, cytological studies showed that only line A17 accumulated autofluorescent soluble compounds, probably flavonoids, in inoculated roots. Such autofluorescent phenolic compounds were also induced by *A. euteiches* in roots of the resistant line A17 and to a lesser degree in the susceptible line F83005.5 (Djébali et al., 2009). Induction of genes involved in phenylpropanoid, terpenoid, and flavonoid biosynthesis is the most common response to *Verticillium* infection in resistant and in susceptible plants (Pegg and Brady, 2002; Fradin and Thomma, 2006). Accumulation of medicarpin, the major isoflavonoid phytoalexin in *Medicago* spp. (Naoumkina et al., 2010), has been observed in alfalfa callus inoculated with *V. albo-atrum* (Latunde-Dada and Lucas, 1986). The antifungal properties of phenolics known to be involved in defense against *Verticillium* wilt (Beckman, 2000; Báidez et al., 2007; Eynck et al., 2009). The phenolics-storing parenchyma cells may release these compounds into the xylem vessels to inhibit pathogen growth.

The resistant line A17 exhibited a higher level of gene expression for the functional category “secondary metabolism” at t0, compared to line F83005.5. This might indicate a state of “preparedness” and allow for a more efficient induction of phenolics after inoculation with *Va* (this study) or *A. euteiches* (Djébali et al., 2009). It has been reported that *Va* and other alfalfa pathogenic fungi are able to metabolize medicarpin (Soby et al., 1996) which might explain that phenolic compounds were not observed in the heavily colonized susceptible line.

### Defense genes are induced in the resistant line after inoculation with *V. alfalfae* whereas the susceptible line suffers profound reprogramming of primary functions

Analysis of the response to *Va* inoculation by GO term enrichment showed that resistant line A17, in addition to genes of secondary metabolism, induced genes related to stress response, and notably to defense such as genes for PR4 protein and BetV1 proteins belonging to the PR10 family (Breiteneder et al., 1989).

In contrast, the susceptible line F83005.5 was severely affected in response to stress, defense mechanisms and in several metabolic pathways. Such a strong effect at early infection stages when only a very small part of the root is colonized, suggests that V31-2 is able to repress defense mechanisms and dysregulate plant metabolism by secreted effectors or toxins. *Verticillium* is described to produce several toxins which induce wilting symptoms but may also act as elicitors of defense, depending on their concentration and/or the plant genotype (Meyer et al., 1994; Wang et al., 2004; Palmer et al., 2005). Preliminary experiments showed that culture filtrate of strain *Va* V31-2 induces wilting symptoms and necrosis in *M. truncatula* (data not shown). SA has been reported to protect cotton callus against *Verticillium* toxin (Zhen and Li, 2004). The SA pathway was down-regulated in the susceptible *M. truncatula* line, notably genes encoding the SA receptor *SABP2* (Kumar and Klessig, 2003) and the SA-regulated glutathione S-transferase (Uquillas et al., 2004), which might lead to enhanced susceptibility to toxins putatively produced by *Va* during infection.

### Co-expression analysis identified a resistance-associated module and suggests that resistance to *V. alfalfae* involves PAMP-triggered immunity

By applying WGCNA using the MACE data of resistant line A17 we identified one module (called Greenyellow) of the co-expression network which was highly related to inoculation response and contained genes encoding LRR-RLKs, NB-LRRs or regulatory elements including transcription factors.

Various gene homologs encoding proteins involved in PAMP perception were detected, including BAK1, EFR, the chitin receptor CERK1, and PRK5 (Miya et al., 2007). Chitinases which release chitin fragments from fungal cell walls (Adams, 2004) were also present in the module. PRK5 encodes a protein kinase receptor with a PR5 family extracellular ligand binding domain (Wang et al., 1996). PR5 specifically binds and hydrolyzes β-1,3-glucans, components of fungi, and oomycete cell walls (Osmond et al., 2001). PRK5 probably fixes the same ligands as PR5. The receptor kinase BAK1 is required for the function of certain Pattern Recognition Receptors (PRR) lacking cytoplasmic signaling domain such as *Ve1* (Heese et al., 2007; Wang et al., 2010; Fradin et al., 2014). BAK1 has been shown to be involved in Arabidopsis resistance to *V. longisporum* (Roos et al., 2014), together with the brassinolide, JA and ABA signaling pathways. BAK1 interacts with the receptor EFR (Heese et al., 2007), which recognizes the bacterial PAMP EF-Tu (Zipfel et al., 2006). Surprisingly, BAK1 was also shown to be involved in the susceptibility of Arabidopsis Col-0 to *V. dahliae* (Gkizi et al., 2016).

Several homologs of genes involved in PTI signaling, such as NDR1, EDR1, and MKK2 (or MEK2) were also present in the resistance-associated module Greenyellow. NDR1 which is required for the activity of several CC-NB-LRR R proteins such as RPM1 (Aarts et al., 1998), participates in ETI and in PTI (Knepper et al., 2011), while EDR1 is a MAP kinase which exerts negative feedback on the activation of MAPK cascades and plays a key role in regulating defense mechanisms (Zhao et al., 2014).

The whole picture suggests that the *M. truncatula* response may resemble innate immunity and PTI rather than the more specific ETI, similar to the situation in resistant tomato and cotton. Indeed, NDR1, BAK1, and MEK2 were found to be required in tomato (Fradin et al., 2009), and FLS2, EFR, CERK1, BAK1, RPM1, PRS2, and MKK2 were found to be induced in resistant cotton *G. raimondii* after inoculation with *V. dahliae* (Chen et al., 2015). However, in *M. truncatula* the response is triggered without implication of a *Ve*1 homolog, suggesting a different mechanism of recognition of *Verticillium* and possibly of signaling.

### Genes in the resistance-associated co-expression module indicate regulation by auxin and abscisic acid

In addition to genes involved in PAMP perception and signal transduction, the Greenyellow module contains genes related to hormone signaling. Although MapMan and GO term enrichment did not show significant induction of these genes in response to inoculation or over-expression at t0 in the resistant line, their co-regulation suggests their involvement in the resistance response. We identified the nodulin MtN21 (a homolog of *Wat1*), and *mlo2* (mildew resistance locus 2 O) in this co-expression module. MLO proteins are involved in resistance against downy mildew in barley but also in dicot plants and prevent pathogen penetration (Consonni et al., 2006; Acevedo-Garcia et al., 2014). MtN21/WAT1 involved in secondary cell wall formation The Arabidopsis *Wat1* mutant is more resistant to several vascular pathogens, probably due to constitutive activation of the SA pathway and inhibition of the auxin pathway (Denancé et al., 2013). Such effects on SA and auxin pathways were also observed in the *mlo2* mutant (Consonni et al., 2006). The expression of these homologs in *M. truncatula* indirectly suggests activation of the auxin pathway in response to *V. alfalfae*.

WGCNA showed that the resistance-related response of line A17 was associated with induction of 21 putative TFs, most of them (71%) being also co-expressed in response to other pathogens of *M. truncatula*. Four among them are involved in ET signaling (Medtr1g069960.1, Medtr1g087920.1, Medtr1g093600.1, Medtr6g037610.1). In addition, genes encoding for ACC oxidases and genes with GO annotations “response to ethylene stimulus” like *EBF1* (EIN3-binding F-box protein 1; Medtr4g114640.1) were identified. This indicates that ET signaling is activated in response to *V. alfalfae*. However, since ET signaling genes were also induced in the susceptible line and ACC pretreatment did only delay disease symptoms, ET does probably not contribute significantly to resistance.

Some TFs of the Greenyellow module belong to the MYB, NAC, bHLH, WRKY, and ERF TFs families that are also the most represented in the cotton transcriptome in response to *V. dahliae* (Zhang Y. et al., 2013). WRKY TFs play a crucial role in defense mechanisms (Pandey and Somssich, 2009), NAC TFs are involved in the response to abiotic stress and pathogens (Nuruzzaman et al., 2013) and bHLH93 is implicated in flowering in *A. thaliana*. Interestingly, flowering time and symptom severity were linked in *V. longisporum*—inoculated Arabidopsis (Häffner et al., 2010). These particular TFs were also found to participate in some ABA responses (Cutler et al., 2010; Wang Y. et al., 2013), and among the five genes encoding for PR10 proteins in this module, three encode for ABA-responsive protein ABR17 with a BetV1 domain. These results together with efficient protection by ABA pretreatment give some indication that the ABA pathway might be involved in *M. truncatula* resistance against *V. alfalfae*.

### Co-expression analysis suggests that *Verticillium* resistance in *Medicago truncatula* may encompass a more general response to pathogens

A large number of genes identified in the resistance-associated Greenyellow module was also found to be co-expressed during the interaction of *M. truncatula* line A17 with four other root pathogens, among them 15 TFs (Figure 5). Because line A17 is susceptible toward three of these other pathogens, co-expression of these genes does not suggest, at first glance, a straightforward and decisive role uniquely in disease resistance. However, the experimental conditions were differing between the pathosystems and the data analyzed usually represent only a snapshot of the whole infection process. As a consequence, differences in intensities and/or timing of gene expression in this composite dataset might still not be sufficient to explain resistance to one given pathogen and susceptibility to others. We advocate that there is probably not one ultimate master switch responsible for the phenotype in QDR, but rather a collaboration of several regulatory genes which contribute to the final result. The core pathogen-responsive genes identified in our study might thus still participate in basal defense of *M. truncatula* against root pathogens.

## Conclusion

Quantitative resistance of *M. truncatula* to *V. alfalfae* V31-2 involves the PTI signature elements NDR1, BAK1, and MKK2, as also reported in tomato and cotton, but does not rely on *Ve*1 or *Ave*1 homologs. The overlapping gene co-expression pattern in *M. truncatula* line A17 in response to *V. alfalfae* and to other root pathogens supports the view that A17 resistance to *Verticillium* presents major traits of PTI. Hence the interaction between *M. truncatula* A17 and *V. alfalfae* V31.2 provides an excellent model to investigate the relationship between PTI and quantitative resistance in a legume plant. Because of the quantitative nature of *Verticillium* resistance in *M. truncatula* and of the high number of putative regulatory and signaling genes with probably subtle and combined effects, a whole-genome approach based on the biodiversity in this plant species seems to be the most appropriate to address this question.

## Author contributions

MT performed most experiments and GO term and MapMan analysis. CB contributed to the experimental design, statistical, and data analysis. AL participated in the microscopy study. LG contributed to the experimental design and performed statistical analysis of the MACE libraries sequencing data and WCGNA. MR contributed to the experimental design and data analysis. MT, CB, LG, and MR wrote the manuscript. All authors read and approved the final manuscript.

### Conflict of interest statement

The authors declare that the research was conducted in the absence of any commercial or financial relationships that could be construed as a potential conflict of interest.

## Abbreviations

DEG: Differentially expressed gene
dpi: days post-inoculation
GO term: Gene ontology term
qPCR: quantitative PCR
qRT-PCR: quantitative Reverse Transcription PCR
TF: Transcription factor
*Va*: *Verticillium alfalfa*.
