# Supplementary material for: Quantitative Resistance to Verticillium Wilt in Medicago truncatula Involves Eradication of the Fungus from Roots and Is Associated with Transcriptional Responses Related to Innate Immunity
Source: Front Plant Sci. 2016 Sep 29;7:1431. doi: 10.3389/fpls.2016.01431 (PMC5041324; doi:10.3389/fpls.2016.01431)
Supplement: Supplementary file 1 [file Table1.pdf]

Supplementary Table S1. List of primers used in this work.

ID Mt v3.5 and ID Mt v4.0 correspond respectively to gene IDs on versions v3.5 and v4.0 of *M. truncatula* genome.

**MACE validation by qRT-PCR**			
**ID Mt v3.5**	**Annotation**	**Forward**	**Reverse**
IMGA_Contig_240964_1.1	wound-responsive family protein	GCAACAAGAGGAGCTTTGGT	GGACCCCATATGCTCAAGAA
IMGA_contig_55783_1.1	ubiquitin ligase SINAT3	CGATTCATGGGTGATGACAG	TCCCCACCTGAGAAGAAAAG
IMGA_contig_70372_1.1	Defensin-like protein	CTTGCCCTTCTCCTTATTTCC	CACACTTTTGCTTCCACCTC
IMGA_contig_73988_1.1	zinc finger, C3HC4 type (RING finger) protein	GGATTCAACCGTCGAGTCAT	TGCTGCTCTGATTCTGGAAC
IMGA_contig_83034_1.1	leucoanthocyanidin dioxygenase-like protein	AGGTGCTGAGCAATGCAA	TCCACAAGGTCCTTTCATCC
Medtr1g083950.1	universal stress family protein	CGGCAACCATGGAGAAGTAT	GTGGCTTCCCATAACCAAGA
Medtr2g035440.1	haloacid dehalogenase-like hydrolase domain protein	GGTTCATCTGCGCCATATAC	GCTGGAAGAATCACATCTCG
Medtr2g099470.1	Chitinase (Class IV) / Hevein	ACGGGTATTGTGGTAATGGTG	GGAGTCATCCAAAGATCCAGAC
Medtr2g101370.1	major intrinsic protein (MIP) family transporter	TTGGAGCTGGTGAAGGAGTT	ATTGACCCACCAGAGAATGG
Medtr3g047140.1	specific tissue protein	ACCCTTATGCGAATGTAGGC	TGAAAGCGGTAGTGTCATCG
Medtr3g089970.1	zinc-binding alcohol dehydrogenase family protein	CACATTTTCCCTCGCTAGTC	ACGCCATGAGATTGCATAC
Medtr4g021260.1	ferretin 1	CGGTGTTTCAGAATGGTACG	CTCCGCCAGTAGGATCAAAT
Medtr4g126920.1	plant invertase/pectin methylesterase inhibitor	GGTTCCTCTTGCTCTTCCAA	GCCTTGAGGAAATCGCTTAG
Medtr5g040430.1	transmembrane protein, putative	GGTGTTTGAAGAAGGGGTCA	TGCTTCGTAGGTGCAAGAAG
Medtr5g089580.1	hypothetical protein	CCTTCTCAAGCCATTCCATC	GTGGATCTTGTGGCTCCATT
Medtr7g016700.1	chalcone and stilbene synthase family protein	TGAGGCTTTTGAGCCATTG	AGCACTTCTCTGGTTGCATTC
Medtr8g022300.1	dormancy/auxin associated protein	ACAGGTTTGGGTAAGCTTCG	GAGGTGTTGTAGCAGCAGAAGA
TC175803	cytochrome P450 family flavone synthase	TGTTGGTGAAGGTGAAGCAG	TATGTGCCCTTGGAACTGTG
TC183087	haloacid dehalogenase-like hydrolase domain protein	TCTCTCTCTCTCTCTCTCTCTCTCAC	AGCCAGAAGCTAAGAAAGCTG
TC191486	F-box SKP2A-like protein	AGCGATGTTGGAGTGATGAG	AGGGACTTTAGATGAGGACACC
Medtr8g018570.1	linoleate 9S-lipoxygenase	GGTCAATGCCGAGGGTATTA	AGGCCTTGCTCAGTGAAAAC
TC179073	jasmonate zim-domain protein	CCTCAGCTTGTGGAAAATCC	GCTAATTTGGTGGCCAACTC

TC176982	glutathione S-transferase, amino-terminal domain protein	GGCAAAATCACTTCCTCACC	CACATAGTTTCTCCTCCGGTTC
TC197452	methyl esterase 1	GTCTTGGAGGACTTAGCATAGCTC	CTGGCAGAATTCAGTGTCCA
Medtr4g100380.1	white-brown-complex ABC transporter family protein	GCGGCTTTGGCTTATGATAG	TGTTGTGCCACTTGACTTCC
Medtr1g093600.1	AP2/ERF and B3 domain transcription factor	TCCAGAAATCTACGGCGTCT	CACGCTGGTGTTTCTCGTAA
Medtr3g109160.1	kinase C-like zinc finger protein	GGAAGGAGGAGGATCAAACA	GAGCCAAATACTCGGTTGGT
Medtr8g022440.1	SAUR-like auxin-responsive family protein	CGTGAACTCCGATGTCATTC	CCCCAATTGTGTCTCACCTT
TC189643	Ethylene-responsive transcription factor	CGTAGAAGACCATGGGGAAA	AAGAAGGTGGTGGAGGCATA
Medtr4g075980.1	Stress-induced transcription factor NAC1	TGTGACTTGCCAGGTTTGTC	TGGTTCCTCTTGGAGCTCTT
Medtr3g111290.1	Transcription factor bHLH 93	CACTGTCCATGATGGGAGAA	GATTCTTCGACGGTTGTCCT
contig_76909_1.1	WRKY transcription factor 28-like	TTCTTCTCCACATGCACCAC	GGGACAATGTCTTGAAGGAGTC
**qRT-PCR normalisation**			
Medtr2g099090.1	hydroxyacylglutathione hydrolase	CAGGTGATTGGTTCAGCAAC	CCGCAAAGTAGAAGCTGATG
Medtr2g033910.1	protein phosphatase 2C family protein	GATGCTGCTCGTTACGTTTG	ACTTGAGGCCGAGTATTCCA
Medtr3g085850.1 *	glyceraldehyde-3-phosphate dehydrogenase	TGCCTACCGTCGATGTTTCAGT	TTGCCCTCTGATTCCTCCTTG
TC117750 **	H3L-like histone	ATTCCAAAGGCGGCTGCATA	CTTTGCTTGGTGCTGTTTAGATGG
***Verticillium* DNA quantification by qPCR**			
**Primer name**		**sequence**	**reference**
vert853F		CGAGTTCGCGGCAGGTA	Larsen et al., 2007
vert927R		GGCCACGCTAGCCTTCACTA	
***Verticillium* species analysis by PCR**			
**Primer name**	**Specificity**	**sequence**	**reference**
Df	*V. dahliae*	CCGGTCCATCAGTCTCTCTG	Inderbitzin et al., 2013
Dr	*V. dahliae*	CTGTTGCCGCTTCACTCG	
AlfD1r	*V. alfalfae*	TGCCGGCATCGACCTTGG	
Alff	*V. alfalfae*	TCATGCCCCCTTTGTTCATCGAT	
NoF	*V. nonalfalfae*	CCTCGAAAAATCCACCAGCTCTA	
NoNuR	*V. nonalfalfae*	GTGGTTGAGATCCTCACGCTTC	
AVe1-F	Race 1	CAGCAATCCCAGCCAATTTCCCTCTG	de Jonge et al., 2012

AVe1-R	Race 1	CTTGCAGGACCCTCTAGCACCACTG	

* used in Ben et al, 2013a; **used in Ariel et al., 2010
