# Supplementary material for: Quantitative Resistance to Verticillium Wilt in Medicago truncatula Involves Eradication of the Fungus from Roots and Is Associated with Transcriptional Responses Related to Innate Immunity
Source: Front Plant Sci. 2016 Sep 29;7:1431. doi: 10.3389/fpls.2016.01431 (PMC5041324; doi:10.3389/fpls.2016.01431)
Supplement: Supplementary file 2 [file Table2.PDF]

Supplementary Table S2. Total number of counts in the twelve MACE libraries.

RB1 and RB2 correspond to two independent experiments; t0 corresponds to root samples harvested just before inoculation with *V. alfalfae*; Early-Mock and Early-*Va* V31-2 correspond to libraries produced from pools of root samples from 4, 8 and 24 hpi for mock-inoculated or inoculated plants, respectively.

***M. truncatula* line**	**Library**	**Total number of counts**
**A17**	**RB1 t0**	29.678.138
	**RB1 Early-Mock**	14.041.789
	**RB1 Early-*Va* V31-2**	18.319.842
	**RB2 t0**	13.084.005
	**RB2 Early-Mock**	20.420.970
	**RB2 Early-*Va* V31-2**	19.028.423
**F83005.5**	**RB1 t0**	17.631.886
	**RB1 Early-Mock**	20.510.566
	**RB1 Early-*Va* V31-2**	14.949.498
	**RB2 t0**	13.976.829
	**RB2 Early-Mock**	14.622.005
	**RB2 Early-*Va* V31-2**	27.193.063
