# Supplementary material for: Quantitative Resistance to Verticillium Wilt in Medicago truncatula Involves Eradication of the Fungus from Roots and Is Associated with Transcriptional Responses Related to Innate Immunity
Source: Front Plant Sci. 2016 Sep 29;7:1431. doi: 10.3389/fpls.2016.01431 (PMC5041324; doi:10.3389/fpls.2016.01431)
Supplement: Supplementary file 6 [file Table6.PDF]

Supplementary Table S6. MACE data validation by qRT-PCR.

A17				
			**Fold change**	
**Gene ID Mt3.5**	**Gene ID Mt4.0**	**Functional annotation**	**MACE**	**qRT-PCR**
Medtr2g035440.1	Medtr2g035440.1	Haloacid dehalogenase-like hydrolase domain containing protein	4.41	3.5
Medtr3g089970.1	Medtr3g089970.1	Alcohol dehydrogenase	3.91	11.01
TC183087	Medtr2g035440.1	Dreg2 like protein	3.84	1.17
Medtr7g016700.1	Medtr7g016700.1	Chalcone synthase	2.74	2.16
TC175803	Medtr4g088195.1	Isoflavone synthase 1	2.74	2.55
Medtr2g099470.1	Medtr2g099470.1	Endochitinase PR4	2.54	2.22
Medtr8g022300.1	Medtr8g022300.1	hypothetical protein	-2.6	-2.8
Medtr5g089580.1	Medtr5g089580.1	hypothetical protein	-3.06	-2.21
Medtr2g101370.1	Medtr2g101370.1	Aquaporin	-3.81	-2.47
TC191486	Medtr1g029500.1	Fbox protein AtFBL5	-4.12	-4.68
contig_70372_1	Medtr8g012795.1	Defensin-like protein	-5.41	-7.14
**F83005.5**				
			**Fold change**	
**Gene ID Mt3.5**	**Gene ID Mt4.0**	**Functional annotation**	**MACE**	**qRT-PCR**
contig_240964_1.1	Medtr2g089835.1	Wound induced protein	4.67	5.96
Medtr8g018570.1	Medtr8g018570.1	Lipoxygenase	3.75	2.18
contig_83034	Medtr7g068650.1	1-aminocyclopropane-1-carboxylate oxidase	2.77	2.46
Medtr1g083950.1	Medtr1g083950.1	Universal stress protein A-like protein	2.71	2.56
Medtr4g126920.1	Medtr4g126920.1	Xyloglucan endotransglucosylase/hydrolase	2.29	1.43
TC179073	Medtr8g021380.1	jasmonate zim-domain protein	2.17	1.46
TC197452	Medtr8g079120.1	Galactose-binding like	-1.62	-1.87
contig_55783	Medtr7g117415.1	E3 ubiquitin protein ligase sina	-2.00	-1.99
Medtr4g021260.1	Medtr4g021260.1	Avr9Cf9	-2.35	-2.59
TC176982	Medtr3g467420.1	glutathione s- amino-terminal domain protein	-2.69	-4.99
Medtr5g040430.1	Medtr5g040430.1	Glutathione S-transferase	-2.86	-2.22
contig_73988_1.1	Medtr2g055790.1	RING finger family protein	-4.46	-2.95
Medtr3g047140.1	Medtr3g047140.1	Lectin	-5.58	-2.34

ID Mt v3.5 and ID Mt v4.0 correspond respectively to gene IDs on versions v3.5 and v4.0 of *M. truncatula* genome. MACE fold change values correspond to the ratio [mean of normalized counts in *Va*-inoculated condition/ mean of normalized counts in mock-inoculated condition] if fold $\geq$1; or to -1/fold if fold <1. *Va: Verticillium alfalfae*. qRT-PCR fold change values were determined using the $\Delta\Delta C_T$ method, using the mock-inoculated control condition as the reference (Livak & Schmittgen, 2001).
