# Supplementary material for: Quantitative Resistance to Verticillium Wilt in Medicago truncatula Involves Eradication of the Fungus from Roots and Is Associated with Transcriptional Responses Related to Innate Immunity
Source: Front Plant Sci. 2016 Sep 29;7:1431. doi: 10.3389/fpls.2016.01431 (PMC5041324; doi:10.3389/fpls.2016.01431)
Supplement: Supplementary file 7 [file Table7.PDF]

Supplementary Table S7. Effect of plant hormones on *Va* V31-2 growth and sporulation *in vitro*.

Growth of fungal colonies was measured at 8 and 15 days of culture at 25°C on PDA medium amended with 10 μM of SA, ABA, IAA, MeJA or ACC. Spore production was determined after 15 days of culture, by flooding the culture plates with 15 ml of sterile water and counting with a hemocytometer. Values are the means ± SD of one experiment with four technical replicates.

Hormone	Colony diameter (mm) ± SD		Conidia (x10^7/ml) ± SD
	8 days	15 days	
Control	43,0 ± 2,6	79,0 ± 1,0	1,02 ± 0,2
SA	41,3 ± 1,2	77,3 ± 0,6	0,97 ± 0,2
ABA	43,0 ± 1,0	79,0 ± 1,0	0,90 ± 0,1
IAA	42,7 ± 1,2	77,0 ± 1,0	0,98 ± 0,3
ACC	42,0 ± 4,0	78,3 ± 4,5	1,07 ± 0,3
MeJA	42,3 ± 2,1	78,7 ± 2,1	1,02 ± 0,1
