# Supplementary material for: Quantitative Resistance to Verticillium Wilt in Medicago truncatula Involves Eradication of the Fungus from Roots and Is Associated with Transcriptional Responses Related to Innate Immunity
Source: Front Plant Sci. 2016 Sep 29;7:1431. doi: 10.3389/fpls.2016.01431 (PMC5041324; doi:10.3389/fpls.2016.01431)

Supplementary Figure S1. Phenotypes of *Medicago truncatula* accessions A17 (resistant) and F83005.5 (susceptible) after root inoculation with *Verticillium alfalfae Va* V31-2 in hydroponic culture.

Ten-day-old plants were root-inoculated with *Va* strain V31-2 (10^6 spores.ml^{-1}) or mock-inoculated with sterile water and maintained in Farhaeus medium. A, Inoculated (V31-2) and control (Mock) plants cultivated in hydroponic culture at 21 dpi. B, Symptom scale from 0 to 4 for disease index scoring. In compatible interactions, the wilting symptoms were visible one week after inoculation and developed gradually starting from the first leaf (score 1). The wilting spread to the second leaf (score 2), then all the leaves were wilted (score 3), leading to the death of the entire plant (score 4). C, Disease progression curve of inoculated A17 and F83005.5 plants. Means and SE values of six independent experiments are presented. D, Fresh weight of shoots at 21 dpi of controls (Mock) and inoculated plants (V31-2) of three independent experiments. Shoot fresh weight is significantly decreased after inoculation in line F83005.5 (*** : *p*-value = 0.001). dpi: days post-inoculation.

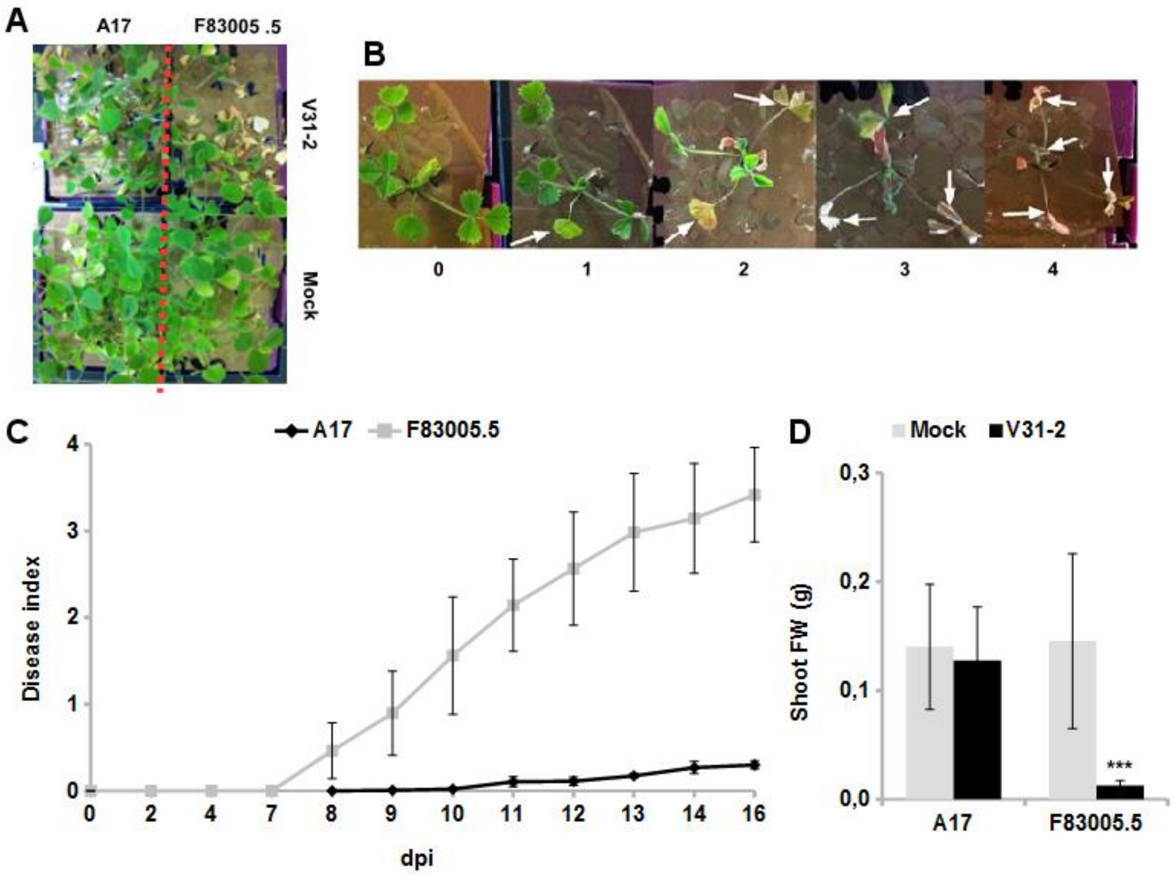

Supplement: Supplementary file 9 [file FigureS1.PDF]
