# Supplementary material for: Quantitative Resistance to Verticillium Wilt in Medicago truncatula Involves Eradication of the Fungus from Roots and Is Associated with Transcriptional Responses Related to Innate Immunity
Source: Front Plant Sci. 2016 Sep 29;7:1431. doi: 10.3389/fpls.2016.01431 (PMC5041324; doi:10.3389/fpls.2016.01431)

Supplementary Figure S2. Autofluorescent compounds in roots of *M. truncatula* lines A17 (resistant) and F83005.5 (susceptible) inoculated with *Va* V31-2, at late stages of infection.

Longitudinal sections of A17 (A) and F83005.5 (B) roots at 7 days post-inoculation were observed under UV light (emission maximum at 365 nm) to visualize autofluorescence of tissues. Autofluorescent compounds are observed in cortex cells of A17 but not in F83005.5. co = cortex ; xy = xylem elements ; f.c = fluorescent compounds.

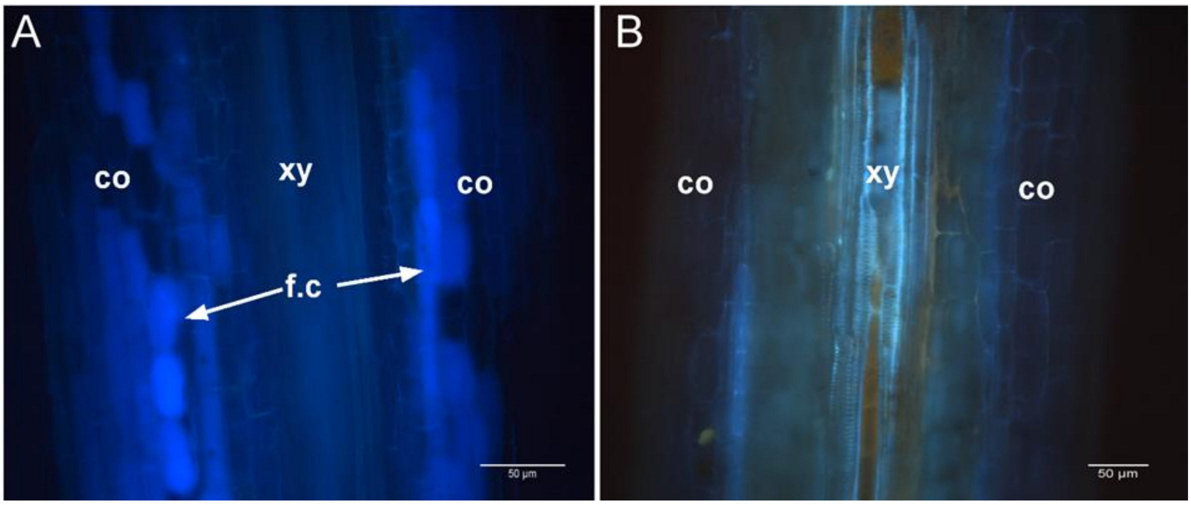

Supplement: Supplementary file 10 [file FigureS2.PDF]
