# Supplementary material for: Quantitative Resistance to Verticillium Wilt in Medicago truncatula Involves Eradication of the Fungus from Roots and Is Associated with Transcriptional Responses Related to Innate Immunity
Source: Front Plant Sci. 2016 Sep 29;7:1431. doi: 10.3389/fpls.2016.01431 (PMC5041324; doi:10.3389/fpls.2016.01431)

Supplementary Figure S3. WGA-FITC staining of roots of A17 (resistant) and F83005.5 (susceptible) inoculated with a GFP-expressing strain of *Va* V31-2.

Longitudinal sections of A17 (A) and F83005.5 (B) roots at 10 days post-inoculation were observed with confocal laser scanning microscopy, after incubation in WGA-FITC (fluorescein isothiocyanate labeled wheat germ agglutinin). Sections were incubated in 100 µg/ml of WGA-FITC (Invitrogen) in phosphate-buffered saline (PBS pH 7.4) for 45 minutes at 37°C, washed twice in PBS for 5 minutes and observed on a glass slide in PBS. Confocal images were acquired with a spectral confocal laser scanning system (SP2 SE, Leica) equipped with an upright microscope (DM 6000, Leica, Germany). The pathogen (arrows) is revealed by green FITC fluorescence in the susceptible line (B) but not in the resistant line (A). co = cortex; hy = hypha; sp = fungal spore ; xy = xylem elements.

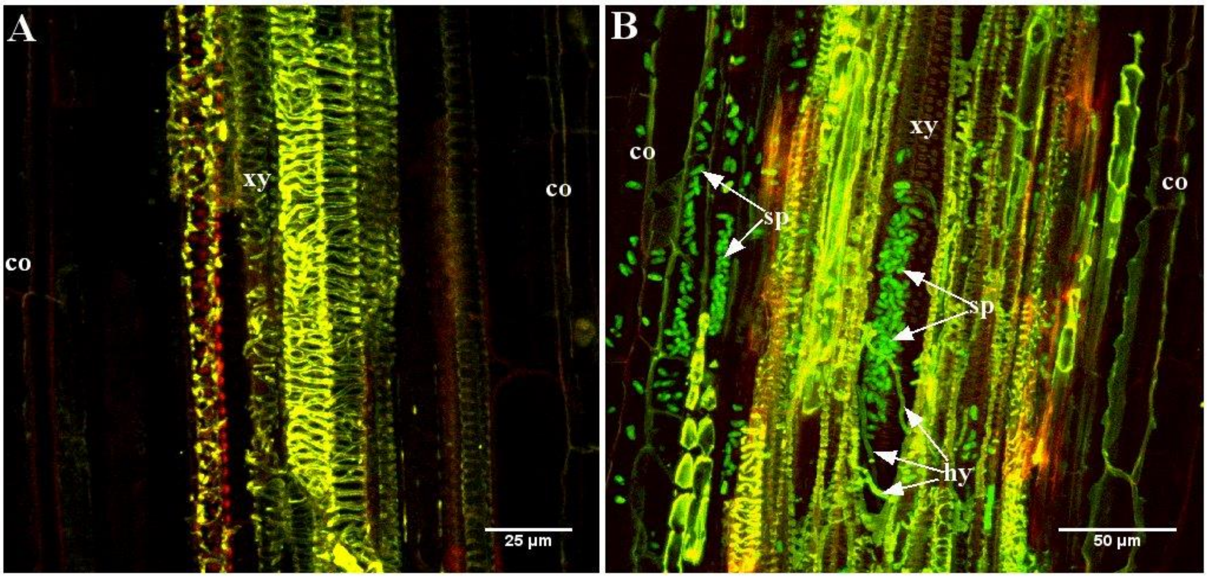

Supplement: Supplementary file 11 [file FigureS3.PDF]
