# Supplementary material for: Quantitative Resistance to Verticillium Wilt in Medicago truncatula Involves Eradication of the Fungus from Roots and Is Associated with Transcriptional Responses Related to Innate Immunity
Source: Front Plant Sci. 2016 Sep 29;7:1431. doi: 10.3389/fpls.2016.01431 (PMC5041324; doi:10.3389/fpls.2016.01431)

Supplementary Figure S4. Root and shoot colonization of *M. truncatula* lines A17 (resistant) and F83005.5 (susceptible) by *Va* V31-2 assessed by relative quantification of fungal DNA.

Verticillium genomic DNA was quantified in roots (A) and aerial parts (B) by quantitative PCR at 7, 10 and 13 dpi. Roots were surface-sterilized 1 min in 1% NaOCl, rinsed thrice for 1 min in water and blotted dry on filter paper (Vicente et al., 2009). Then root systems and aerial parts were separated and dried at 70°C. Total DNA was extracted as described by Murray *et al. (1980)*. DNA was diluted to 50 ng/µl for V31-2 quantification and to 5 ng/µl for *M. truncatula* quantification. Quantitative PCR was performed in optical 384-well plates containing 5 µl of Power SYBR® Green PCR Master Mix reagent (Applied Biosystems), 3 µl of DNA and 5 µM of each primer (total volume 10 µl). Primers are shown in Supplementary Table S1. All reactions were performed in duplicate in an ABI PRISM® 7900 HT Sequence Detection System (Applied Biosystems) with the following protocol: 50°C for 2 min; 95°C for 10 min; 40 cycles of 95°C for 15 sec and 60°C for 1 min. Standard curves were made with 10-fold dilutions of V31-2 purified DNA (from 0.1 ng to 10 ng gDNA, $R^2 \geq$ 0,997) and 2-fold dilutions of F83005.5 purified DNA (from 6.5 ng to 100 ng, $R^2 \geq$ 0,998). Fungal and plant DNA quantities were calculated by converting the C_T value according to standard curves using the SDS 2.3 software (Applied Biosystems).

In the susceptible line, fungal DNA could be detected in roots after 7 days, just before the appearance of symptoms, and in aerial parts after 10 days, and the amount increased steadily thereafter. In plants of the resistant line, fungal DNA was not detected at significant levels neither in roots nor in aerial parts. Data shown are mean values of two independent experiments (3 plants per time point per experiment) ± SE,and are expressed in ng of fungal genomic DNA for 1µg of *M. truncatula* genomic DNA [(ng *Va* DNA)/ (µg *Mt* DNA)]. dpi: days post-inoculation.

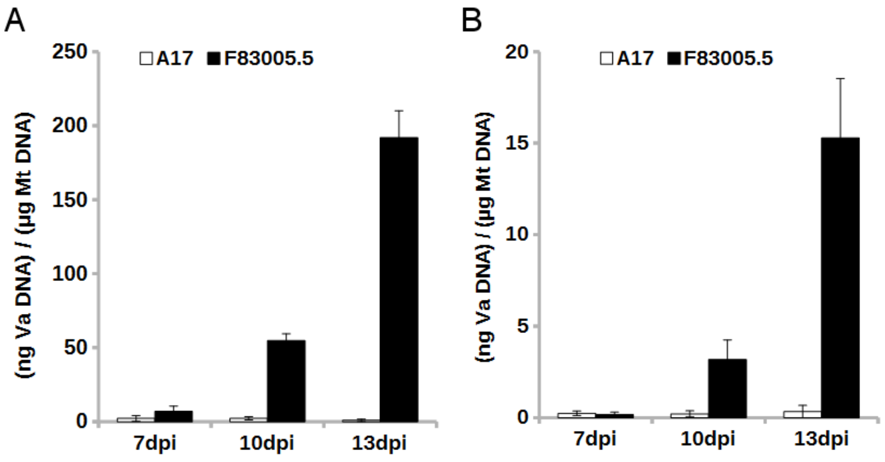

Supplement: Supplementary file 12 [file FigureS4.PDF]
