# Supplementary material for: Quantitative Resistance to Verticillium Wilt in Medicago truncatula Involves Eradication of the Fungus from Roots and Is Associated with Transcriptional Responses Related to Innate Immunity
Source: Front Plant Sci. 2016 Sep 29;7:1431. doi: 10.3389/fpls.2016.01431 (PMC5041324; doi:10.3389/fpls.2016.01431)

Supplementary Figure S5. Molecular characterization of *Verticillium* strain V31-2 by PCR with species- (A) and race-specific (B) primers.

20ng of fungal genomic DNA were amplified with primer pairs *Vd* (Df/Dr) specific to *V. dahliae*, *Va* (AlfF/AlfD1r) specific to *V. alfalfae*, *Vna* (NoF-NoNuR) specific to *V. nonalfalfae* as described by Inderbitzin *et al.* (2013) and Ave1 specific to race 1 (de Jonge et al., 2012). For comparison, DNA from JR2 (race 1) and LPP0323 were included in the experiment. L : 2-log DNA ladder. Lane 1: JR2. Lane 2: V31-2. Lane 3: LPP0323.

Each PCR reaction comprised 40ng of DNA, 2.5µl of 10x PCR buffer, 1.25µl of $MgCl_2$ (50mM), 4µl of dNTP (1.25mM), 0.75 U of Taq polymerase, and 2µl of primer mix (5µM) in a final volume of 25µl. The PCR program for *Va* (AlfF/AlfD1r), *Vna* (NoF/NoNuR) and *Vd* (Df/Dr) was as described by Inderbitzin *et al.* (2013) except that annealing temperature of *Vd* (Df/Dr) was 55°C. For Ave1 amplification, the PCR program consisted of a 2min initial denaturation step at 94°C, 38 cycles of 10 sec at 94°C, 20 sec 62°C, and 1 min at 72°C, followed by a final extension of 7 min at 72°C. PCR products were visualized by electrophoresis in a 1.5 % agarose gel and staining with Ethidium bromide.

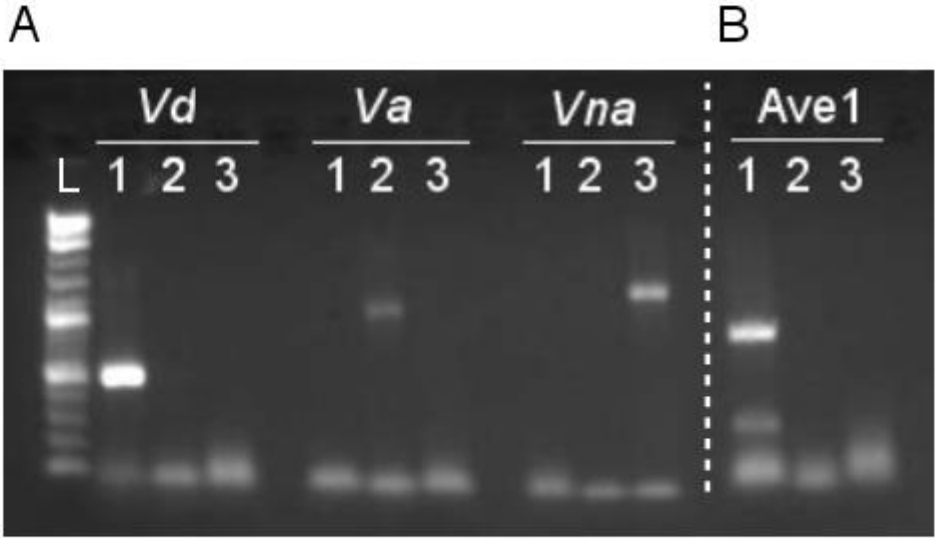

Supplement: Supplementary file 13 [file FigureS5.PDF]
