# Supplementary material for: Quantitative Resistance to Verticillium Wilt in Medicago truncatula Involves Eradication of the Fungus from Roots and Is Associated with Transcriptional Responses Related to Innate Immunity
Source: Front Plant Sci. 2016 Sep 29;7:1431. doi: 10.3389/fpls.2016.01431 (PMC5041324; doi:10.3389/fpls.2016.01431)

Supplementary Figure S6. Evolution of disease symptoms in *M. truncatula* line F83005.5 in the two independent experiments used for transcriptomic studies.

Lines A17 (resistant, no symptoms) and F83005.5 (susceptible) were root-inoculated with strain *Va* V31-2 and disease symptoms rated regularly on a scale from 0 to 4. Line A17, which did not show any wilting symptoms during the two independent experiments, is not represented in this figure. Symptom scores of individual plants (grey lines) were computed to give a modelised disease progress empirical curve (black line) based on a logistic model as previously detailed in Ben *et al.*(2013a). The logistic curves were then fitted to disease index data based on the overall methodology described in Gilligan (1990). Two independent experiments (RB1, RB2) were performed. Time to reach 50% of maximum symptoms was 13 and 14 days post-inoculation, respectively and maximum symptom score at the end of the experiment was nearly 4 in both experiments, showing that plant's responses in both experiments were very similar.

Gilligan CA (1990) Comparison of disease progress curves. New Phytologist 115: 223–242.

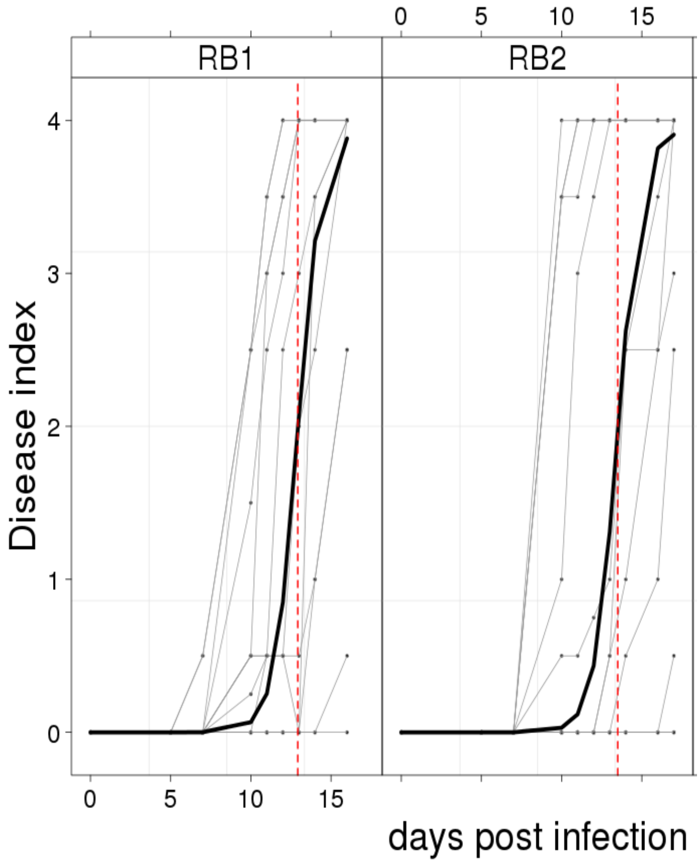

Supplement: Supplementary file 14 [file FigureS6.PDF]
