# Supplementary material for: Quantitative Resistance to Verticillium Wilt in Medicago truncatula Involves Eradication of the Fungus from Roots and Is Associated with Transcriptional Responses Related to Innate Immunity
Source: Front Plant Sci. 2016 Sep 29;7:1431. doi: 10.3389/fpls.2016.01431 (PMC5041324; doi:10.3389/fpls.2016.01431)
Supplement: Supplementary file 15 [file FigureS7.PDF]

Supplementary Figure S7. MapMan 'Biotic stress overview' maps of the Differentially Expressed Genes responding to *Va*-inoculation in the resistant line A17 (A) and susceptible line F83005.5 (B).

Main panel coloured with dark grey gathers genes with experimental indication of involvement in the biotic stress, while left and right sides coloured in light grey group genes and pathways that are putatively involved in biotic stress pathway (Rotter *et al.*, 2007). Each transcript is displayed as a square, the fold change in gene expression for *Va*-inoculated *vs* Mock-inoculated conditions is reported according to a color code with red for up- and green for down-regulated genes after inoculation. *Va: Verticillium alfalfae*.

Rotter A, Usadel B, Baebler S, Stitt M, Gruden K (2007) Adaptation of the MapMan ontology to biotic stress responses: application in solanaceous species. Plant Methods 3:10.

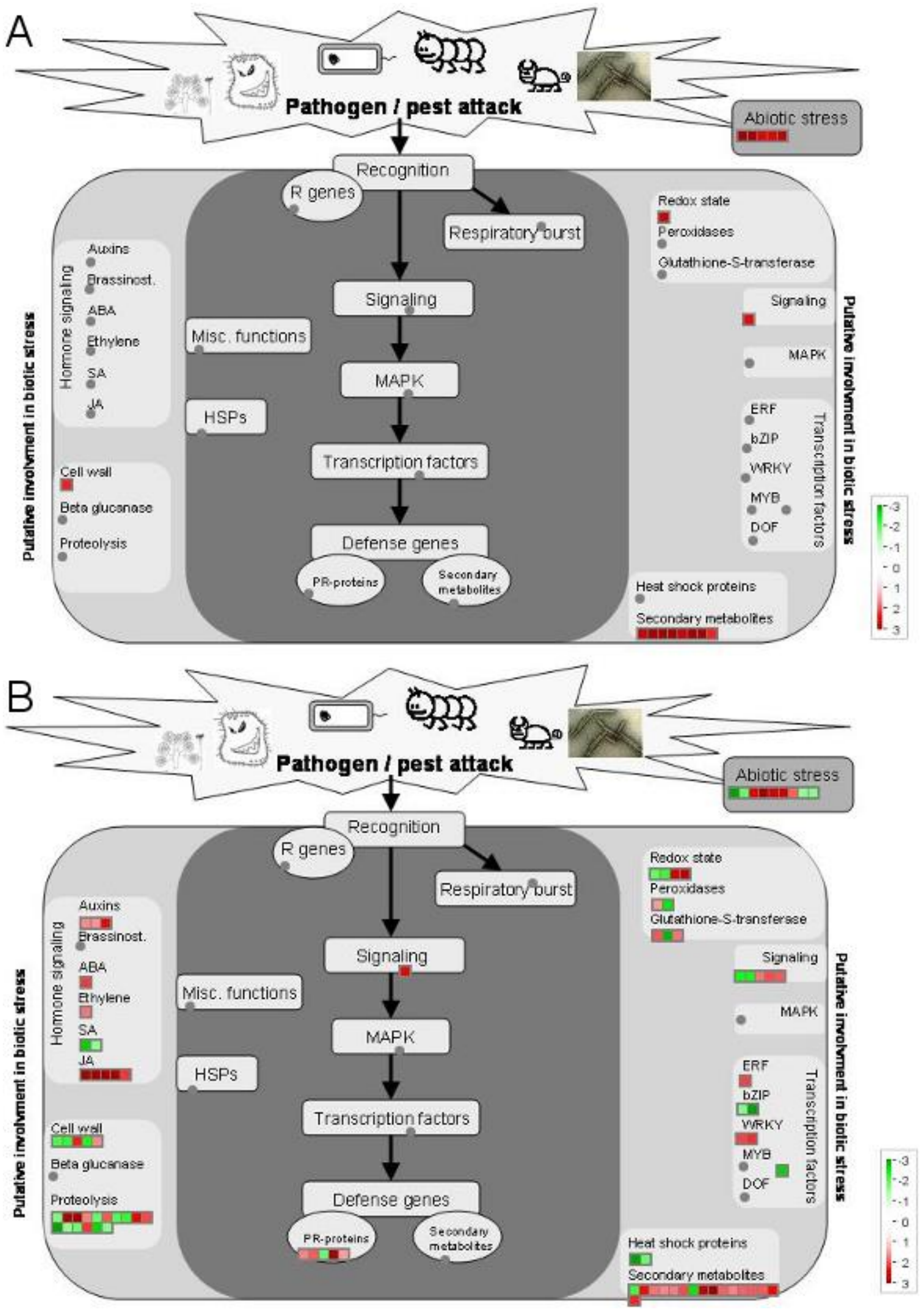
A
Pathogen / pest attack
Abiotic stress
Recognition
R genes
Respiratory burst
Signaling
Misc. functions
MAPK
HSPs
Transcription factors
Defense genes
PR-proteins
Secondary metabolites
Putative involvment in biotic stress
Hormone signaling
Auxins
Brassinost.
ABA
Ethylene
SA
JA
Cell wall
Beta glucanase
Proteolysis
Redox state
Peroxidases
Glutathione-S-transferase
Signaling
MAPK
ERF
bZIP
WRKY
MYB
DOF
Transcription factors
Putative involvment in biotic stress
Heat shock proteins
Secondary metabolites
-3
-2
-1
0
1
2
3
B
Pathogen / pest attack
Abiotic stress
Recognition
R genes
Respiratory burst
Signaling
Misc. functions
MAPK
HSPs
Transcription factors
Defense genes
PR-proteins
Secondary metabolites
Putative involvment in biotic stress
Hormone signaling
Auxins
Brassinost.
ABA
Ethylene
SA
JA
Cell wall
Beta glucanase
Proteolysis
Redox state
Peroxidases
Glutathione-S-transferase
Signaling
MAPK
ERF
bZIP
WRKY
MYB
DOF
Transcription factors
Putative involvment in biotic stress
Heat shock proteins
Secondary metabolites
-3
-2
-1
0
1
2
3

.
