# Supplementary material for: Quantitative Resistance to Verticillium Wilt in Medicago truncatula Involves Eradication of the Fungus from Roots and Is Associated with Transcriptional Responses Related to Innate Immunity
Source: Front Plant Sci. 2016 Sep 29;7:1431. doi: 10.3389/fpls.2016.01431 (PMC5041324; doi:10.3389/fpls.2016.01431)
Supplement: Supplementary file 16 [file FigureS8.PDF]

Supplementary Figure S8. MapMan 'secondary metabolism overview' maps of the Differentially Expressed Genes responding to *Va*-inoculation in the resistant line A17 (A) and the susceptible line F83005.5 (B).

Each transcript is displayed as a square, the log2 fold of expression ratio (inoculated *vs* mock-inoculated) is reported according to the color code shown in the figures. Red is for up- and green for down-regulated genes.

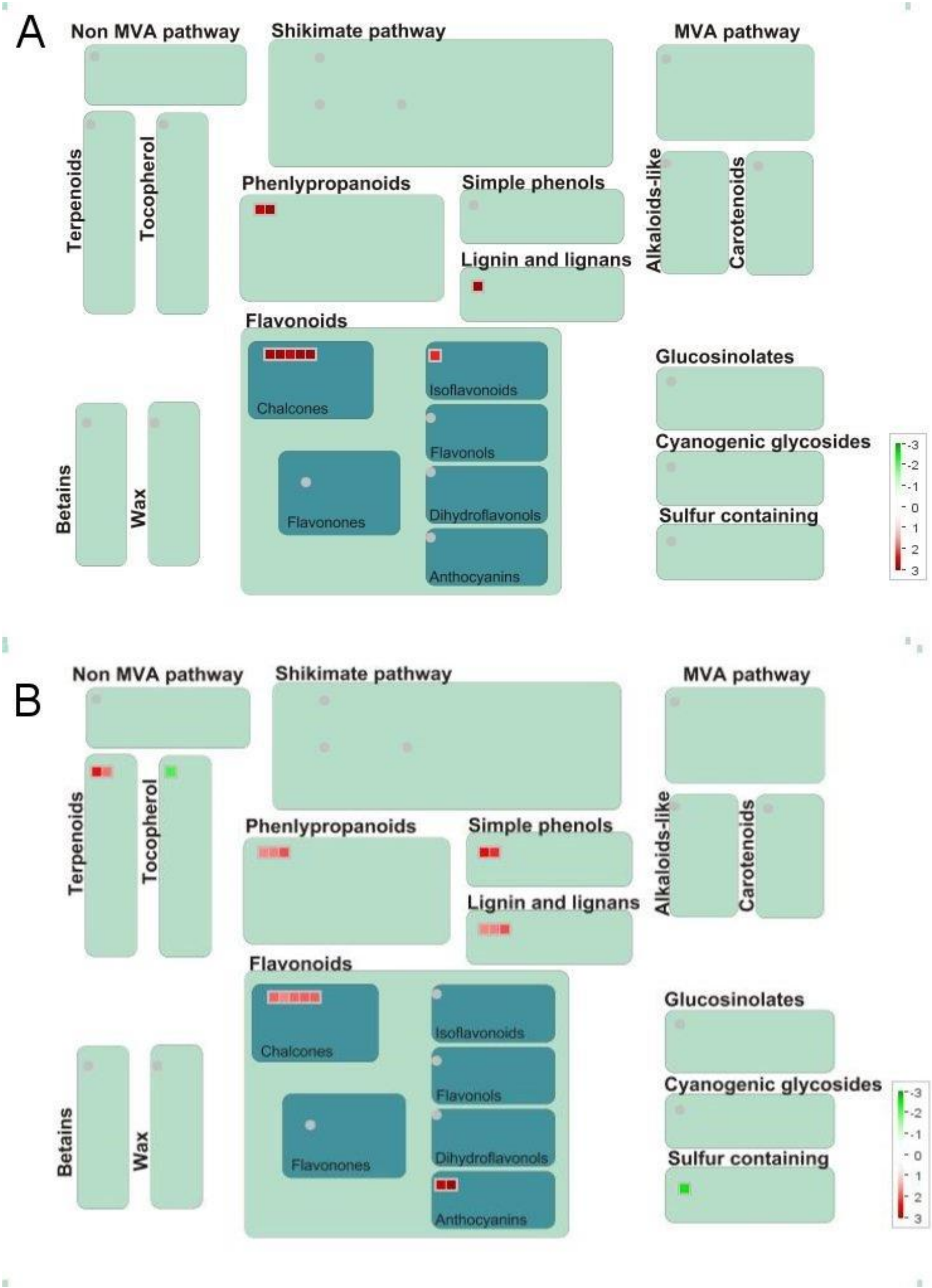
A
Non MVA pathway
Shikimate pathway
MVA pathway
Terpenoids
Tocopherol
Phenlypropanoids
Simple phenols
Lignin and lignans
Alkaloids-like
Carotenoids
Flavonoids
Chalcones
Isoflavonoids
Flavonols
Flavonones
Dihydroflavonols
Anthocyanins
Glucosinolates
Cyanogenic glycosides
Sulfur containing
Betains
Wax
-3
-2
-1
0
1
2
3
B
Non MVA pathway
Shikimate pathway
MVA pathway
Terpenoids
Tocopherol
Phenlypropanoids
Simple phenols
Lignin and lignans
Alkaloids-like
Carotenoids
Flavonoids
Chalcones
Isoflavonoids
Flavonols
Flavonones
Dihydroflavonols
Anthocyanins
Glucosinolates
Cyanogenic glycosides
Sulfur containing
Betains
Wax
-3
-2
-1
0
1
2
3
