# Supplementary material for: Quantitative Resistance to Verticillium Wilt in Medicago truncatula Involves Eradication of the Fungus from Roots and Is Associated with Transcriptional Responses Related to Innate Immunity
Source: Front Plant Sci. 2016 Sep 29;7:1431. doi: 10.3389/fpls.2016.01431 (PMC5041324; doi:10.3389/fpls.2016.01431)
Supplement: Supplementary file 17 [file FigureS9.PDF]

**Supplementary Figure S9. Effect of plant hormone pre-treatments on symptom development induced by *Va* V31-2**.

Nine-day-old plants of the resistant line A17 (A) or the susceptible line F83005.5 (B) were transferred into Farhaeus medium containing 10 µM of ABA (Sigma), ACC (MP Biomedicals), IAA (Sigma), MeJA (Sigma), or SA (Sigma). After 24 hours the plants were inoculated as described in Material & Methods. The hormones remained in the medium until the end of the experiment. Disease symptoms in controls and hormone pre-treated plants were monitored from 0 to 16 dpi. The curves represent the mean disease symptom scores of three independent experiments. Means with the same letter do not differ significantly at $P \leq 0.001$. C = control, SA = salicylic acid, ABA = abscisic acid, ACC = 1-aminocyclopropane-1-carboxylic acid, IAA = indole-3-acetic acid, MeJA = methyl jasmonate. dpi: days post-inoculation.

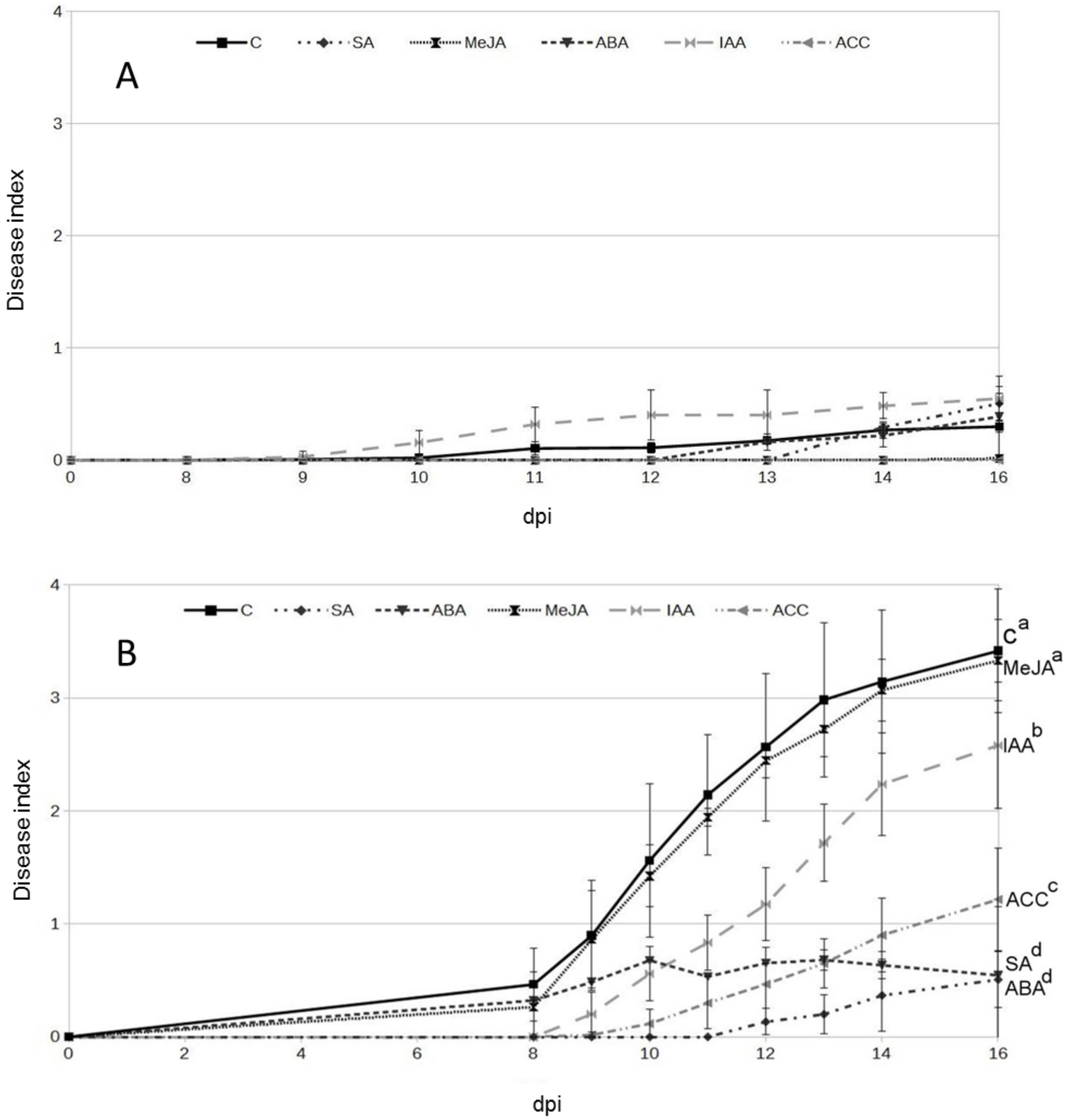
A
B
C
SA
MeJA
ABA
IAA
ACC
Disease index
dpi
C[a]
MeJA[a]
IAA[b]
ACC[c]
SA[d]
ABA[d]
