# Supplementary material for: Quantitative Resistance to Verticillium Wilt in Medicago truncatula Involves Eradication of the Fungus from Roots and Is Associated with Transcriptional Responses Related to Innate Immunity
Source: Front Plant Sci. 2016 Sep 29;7:1431. doi: 10.3389/fpls.2016.01431 (PMC5041324; doi:10.3389/fpls.2016.01431)
Supplement: Supplementary file 18 [file FigureS10.PDF]

Supplementary Figure S10. Effects of hormone pre-treatment on root and shoot colonisation of the susceptible *M. truncatula* line F83005.5 by *Va* V31-2 assessed by relative quantification of fungal DNA.

Fungal genomic DNA was quantified by quantitative PCR in roots (**A**) and aerial parts (**B**) of control (**C**) and pre-treated plants harvested after 7, 10 and 13 dpi. DNA extraction and qPCR were performed as described in legend of Supplementary Figure S4, primers are detailed in Supplementary Table S1. Fungal DNA was strongly reduced in aerial parts of all hormone pre-treated plants compared to controls (*p-value* < 0.001), whereas the effect on root colonization depended on the plant hormone used for pre-treatment. SA and ABA which had the strongest protective effect, also strongly reduced fungal colonization of both roots and shoots. ACC which protects until 16 dpi as efficiently as SA and ABA, reduced colonization of roots and shoots up to 10 dpi (p-value <0.05), but at 13 dpi, roots were colonized as much as in non-treated plants and colonization of aerial parts was also increased though not as high as in non-treated plants. IAA slightly reduced root colonization (p-value<0.001), but less efficiently as SA and ABA, whereas shoot colonization appeared inhibited. Finally, MeJA did not inhibit root colonization but rather stimulated it at early time points. However, shoot colonization was significantly reduced, notably at 13 dpi (p-value <0.001).

Means values of two independent experiments ± SE are represented. Means with the same letter do not differ significantly at $P \leq 0.001$. C = Control, MeJA = Methyl Jasmonate, IAA = indole-3-acetic acid, ACC = 1-aminocyclopropane-1-carboxylic acid, SA = salicylic acid, ABA = abscisic acid, dpi: days post-inoculation.

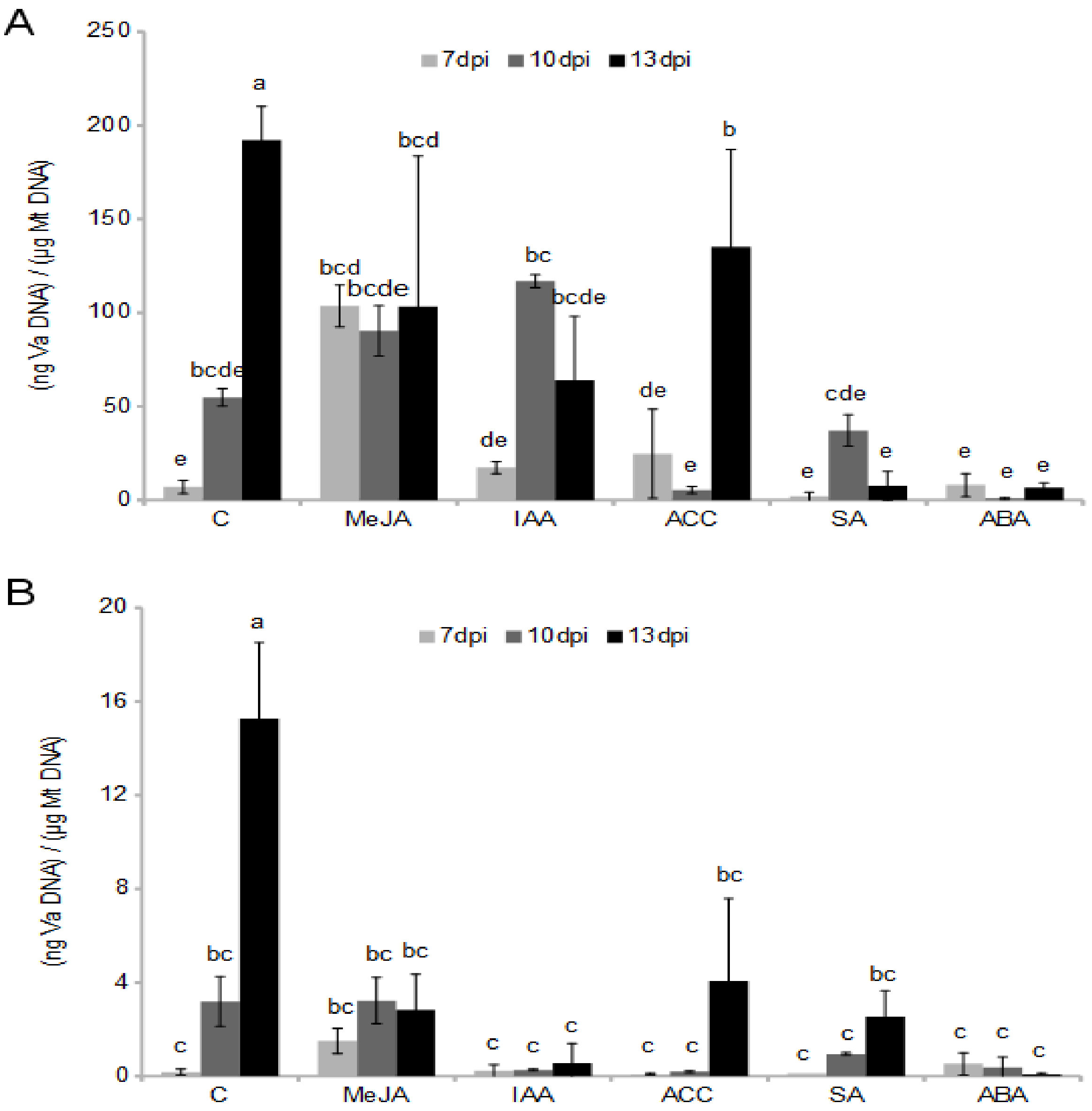

A
250
200
150
100
50
0
7dpi
10 dpi
13 dpi
(ng Va DNA) / (µg Mt DNA)
a
bcd
bcd
bcde
bc
bcde
b
bcde
de
de
e
cde
e
e
e
e
e
e
C
MeJA
IAA
ACC
SA
ABA
B
20
16
12
8
4
0
7dpi
10 dpi
13 dpi
(ng Va DNA) / (µg Mt DNA)
a
bc
bc
bc
bc
bc
bc
c
c
c
c
c
c
c
c
c
c
c
C
MeJA
IAA
ACC
SA
ABA

.
